# Cell identity competes with surface tension to determine stromal organization in tumor spheroids beyond the CAF shell paradigm

**DOI:** 10.1073/pnas.2624455123

**Published:** 2026-10-12

**Authors:** Claire Wilhelm

**Affiliations:** ^a^ Physics of Cells and Cancer, CNRS UMR168, Institut Curie, Sorbonne Université, PSL Research University, Paris 75005, France

**Keywords:** cancer spheroids, cancer-associated fibroblasts, surface tension, magnetic force tensiometry, epithelial-mesenchymal transition

## Abstract

Cancer-associated fibroblasts (CAFs) are often found forming a peripheral stromal barrier around tumors. Here, this paradigm is challenged using microfabricated 3D cancer–CAF spheroid models combined with quantitative magnetic force tensiometry. A surface tension atlas is established for eight cancer cell lines, three CAF populations, and heterotypic tumor–CAF assemblies, spanning nearly two orders of magnitude. Surface tension is first shown to govern spheroid morphology and modulate drug response. Besides, CAFs consistently occupy the highest end of the surface tension hierarchy, predicting core rather than peripheral localization and directly challenging the shell paradigm. Yet confocal imaging reveals not only central accumulation but also complete CAF-tumor mixing, including in spheroids where cancer cells exhibit surface tensions nearly two orders of magnitude lower than CAFs. Surface tension alone therefore fails to predict CAF organization. Instead, the epithelial-to-mesenchymal transition (EMT) state of cancer cells emerges as a major determinant: epithelial-like tumors promote CAF core accumulation, whereas mesenchymal-like states favor persistent mixing. Peripheral CAF localization emerges only under restricted conditions combining high tumor cohesion and sequential stromal recruitment. Together, these results identify surface tension, EMT state, and assembly chronology as joint determinants of tumor–stroma architecture, revealing a context-dependent organization landscape rather than a universal CAF shell.

## Significance

Cancer-associated fibroblasts (CAFs) are widely assumed to form a peripheral stromal shell around solid tumors, yet their spatial organization remains poorly understood. Using magnetic surface tensiometry and engineered tumor spheroids, this study shows that CAF organization is governed not by surface tension alone, but by its interplay with epithelial-to-mesenchymal transition state and assembly chronology. These findings challenge the concept of a universal stromal shell and establish CAF organization as a context- and history-dependent property of tumor architecture.

Cancer-associated fibroblasts (CAFs) have emerged as central regulators of tumor progression and therapeutic response across most solid cancers (1–5). Initially viewed as passive stromal components, CAFs are now recognized as active organizers of the tumor microenvironment, controlling extracellular matrix remodeling, tissue stiffening, angiogenesis, immune regulation, metastasis, and therapy resistance (2, 4, 6–10). CAF-rich stromal regions may also impair drug penetration and contribute to chemoresistance through biochemical and physical mechanisms (6, 8, 9, 11, 12). CAF-mediated matrix deposition can restrict molecular diffusion, while CAF-secreted cytokines and growth factors promote survival signaling and adaptive resistance in tumor cells (7, 9, 13). CAFs have therefore become major therapeutic targets in oncology.

Alongside this functional importance, a remarkably consistent spatial representation of CAF organization has emerged. CAFs are frequently described at the tumor invasive front or as peripheral stromal structures surrounding tumor masses (14–17), under related concepts including *fibrotic capsule*, *stromal shell*, *desmoplastic rim*, or *CAF barrier* (15, 16, 18). Histopathological analyses report fibroblast enrichment at tumor borders (14), while mechanobiological studies suggest that CAF-rich peripheral regions mechanically shield tumors and contribute to immune exclusion (16, 19). More recently, CAF capsules have been described as active mechanical structures capable of compressing tumors and shaping cancer cell behavior (15).

Importantly, however, this consensus largely relies on partial spatial observations. CAF localization is often inferred from two-dimensional histological sections, analyses focused on invasive margins, or systems in which fibroblasts are already present in the surrounding stroma and interact with tumors secondarily (14, 17). Systematic investigation in controlled 3D systems of whether CAFs spontaneously organize at the tumor periphery, whether this is universal across tumor types, or whether it emerges from general physical principles of multicellular organization remains limited.

Among the physical parameters controlling tissue architecture, surface tension occupies a singular position. Originally introduced in developmental biology through Steinberg’s Differential Adhesion Hypothesis (20, 21), tissue surface tension describes the effective cohesion of multicellular assemblies arising from the balance between cell–cell adhesion, cortical tension, and collective mechanical interactions (22, 23). Differential surface tensions have long been proposed to drive tissue segregation, cell sorting, and spatial organization during morphogenesis (20, 21, 24, 25). More broadly, surface tension governs the shape stability, compaction, and organization of multicellular systems, analogously to liquid-like materials (24, 26).

In cancer, multicellular tumor spheroids have become established as physiologically relevant models recapitulating essential aspects of tumor architecture and cell–cell interactions (27–35), providing a platform to investigate how physical principles govern multicellular organization. High surface tension should favor stronger cohesion, greater compaction, and central positioning of more cohesive stromal components, whereas lower surface tension populations should occupy more external or dispersed regions. Surface tension therefore offers a physical framework for understanding how cancer cells and CAFs organize within multicellular tumors. Supporting this view, recent work demonstrated that differential surface tension alone can drive spontaneous core–shell segregation in engineered 3D tissues, with the more cohesive population localizing to the core (36). This raises a fundamental question: do CAFs exhibit higher surface tension than cancer cells, consistent with their contractility and cohesive behavior, and should they therefore localize at the spheroid core rather than the periphery, challenging the prevailing view of CAFs as peripheral stromal structures?

Recent advances in magnetic tissue engineering and biophysics have enabled the remote application of controlled forces to multicellular systems (37–42). Here, magnetic forces are used to compress tumor spheroids under conditions equivalent to a supergravity of approximately 100 g, providing access to their effective surface tension. Beyond this technical development, the primary objective is to systematically interrogate breast, pancreatic, and melanoma cancer cell lines together with corresponding CAFs, establishing the surface tension of heterotypic tumor–CAF spheroids. This large-scale characterization is then confronted with spheroid morphology and chemotherapeutic response, revealing previously unreported correlations between tissue surface tension, multicellular architecture, and drug response. CAFs consistently fall within the upper surface tension range, reaching 60 mN/m, whereas the cancer cell lines analyzed here span 0.1 to 50 mN/m. The 3D organization of CAFs was then examined. When both populations were mixed simultaneously from the outset, no peripheral (OUT) configuration was observed, despite the expectations from surface tension considerations.

However, CAF organization could not be fully explained by the surface tension hierarchy alone. Depending on cellular context, CAFs either localized at the spheroid core or remained homogeneously interspersed with tumor cells. When tumor spheroids were allowed to form before CAF introduction, CAFs again did not systematically form a peripheral layer: they either accumulated at the core or remained mixed with cancer cells. Only under two specific conditions, corresponding to high tumor surface tension, did structured peripheral (OUT) organization emerge, as adjacent dumbbell-like assemblies or, in one instance, an effective CAF shell. Remarkably, CAF organization ultimately correlated with the epithelial-to-mesenchymal transition state of tumor cells, except in the two conditions involving sequential CAF introduction.

Overall, these findings demonstrate that CAF spatial organization is not a universal stromal architecture but emerges from the interplay between surface tension, tumor differentiation state, and the temporal sequence of stromal recruitment during tumor formation.

## Results

### CAFs Define the High End of the Tumor Surface Tension Spectrum.

To quantitatively probe tumor surface tension, a panel of multicellular spheroids spanning epithelial and mesenchymal cancer states across breast, pancreatic, and melanoma cancers was assembled, including four breast cancer cell lines (MCF7, T47D, Hs578T, MDA-MB-231), three pancreatic cancer cell lines (MiaPaCa, PANC-1, hPAF), and one melanoma cell line (A375), together with corresponding cancer-associated fibroblasts. The breast cancer panel includes the epithelial, hormone-dependent lines MCF7 and T47D, the mesenchymal-like TNBC line Hs578T, and the more extreme mesenchymal MDA-MB-231. The pancreatic lines span distinct differentiation states, with MiaPaCa and PANC-1 displaying mesenchymal traits and hPAF retaining epithelial organization. A375 represents a highly mesenchymal and invasive melanoma state. CAFs were included as a common stromal component across tumor types, representing activated fibroblastic populations known to remodel extracellular matrix and modulate tissue mechanics and tumor cell organization.

**Fig. 1.**
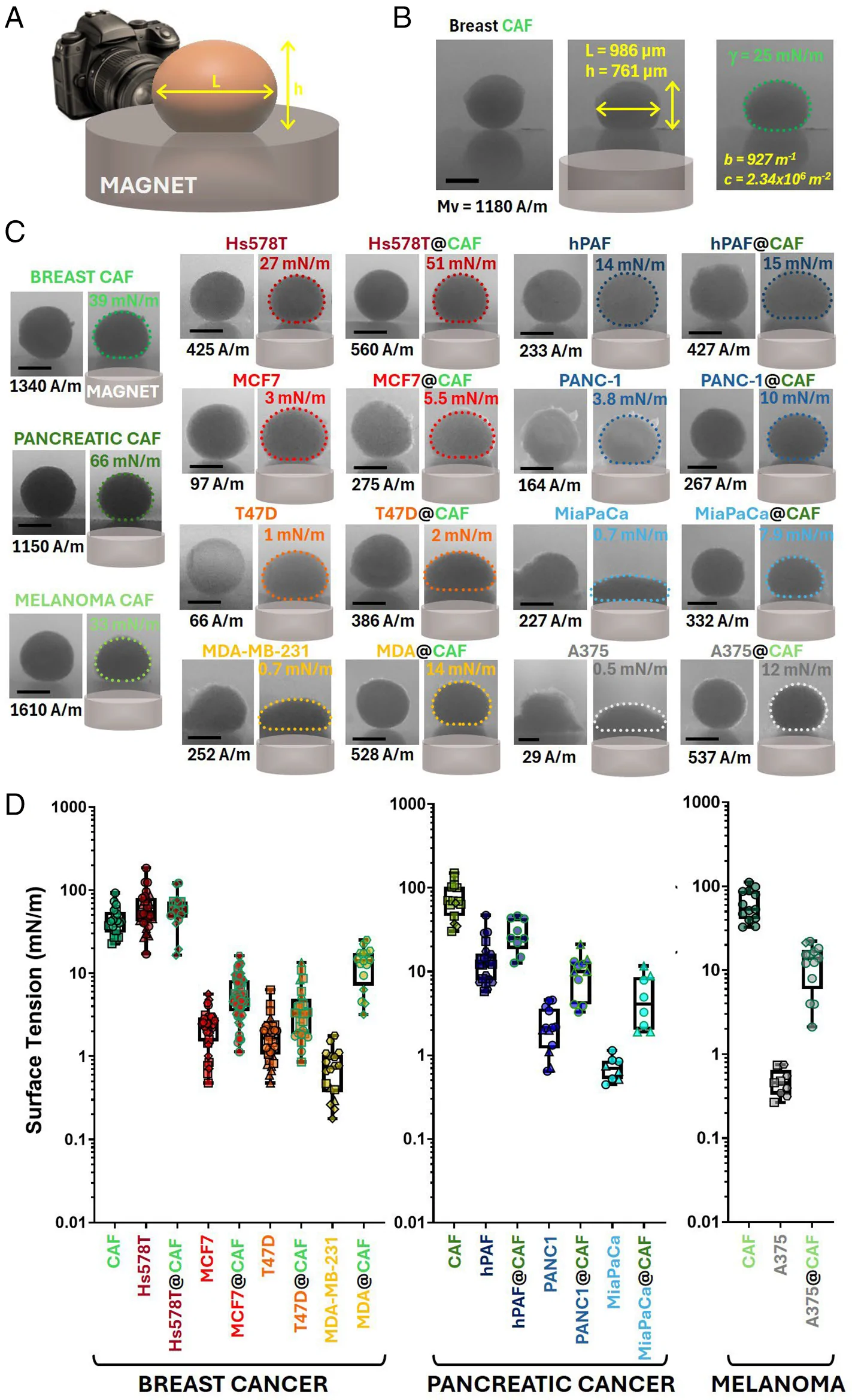
Magnetic tensiometry of tumor spheroids across cancer types and CAF-containing conditions. (*A*) Schematic of the magnetic force tensiometer. Magnetically labeled cells are assembled into 3D multicellular spheroids in nonadhesive agarose microwells and transferred to a temperature-controlled chamber above a permanent magnet generating a uniform magnetic field gradient. The resulting volumetric magnetic body force induces controlled axisymmetric spheroid flattening, with nonwetting boundary conditions, allowing effective interfacial tension to be determined from the balance between magnetic stresses and tissue cohesion. (*B*) Data analysis workflow for effective surface tension determination. Equilibrium side-view spheroid profiles are fitted using a Laplace-based axisymmetric model implemented in MATLAB to extract curvature and capillary constant parameters (*B* and *C*). Combined with independently measured volumetric magnetization, these parameters yield effective tissue surface tension in mN/m. (*C*) Representative equilibrium deformations and corresponding surface tension values for the 19 experimental conditions, including CAFs, tumor cell lines, and 70:30 tumor–CAF mixtures. (*D*) Quantification of effective surface tension across all conditions. Each point represents an independent spheroid. More than 260 measurements were obtained, with the following numbers of independent spheroids per condition: Breast CAF (n = 22), Melanoma CAF (n = 13), Pancreatic CAF (n = 15), Hs578T (n = 29), Hs578T + CAF (n = 17), MCF7 (n = 33), MCF7 + CAF (n = 51), T47D (n = 34), T47D + CAF (n = 25), MDA-MB-231 (n = 18), MDA-MB-231 + CAF (n = 16), A375 (n = 10), A375 + CAF (n = 13), hPAF (n = 22), hPAF + CAF (n = 9), PANC-1 (n = 13), PANC-1 + CAF (n = 11), MiaPaCa (n = 8), and MiaPaCa + CAF (n = 8). The full dataset and statistical analysis are provided in the Source Data file (sheets “Fig. 1” and “Fig. 1-stat”). Statistical analysis was performed on log10-transformed surface tension values using one-way ANOVA with multiple comparisons. Biologically relevant comparisons within each cancer type are reported in *SI Appendix*, Table S2 for breast cancer and *SI Appendix*, Table S3 for pancreatic cancer, melanoma, and CAF-to-CAF comparisons.

Cellular populations were rendered magnetically responsive through internalization of biocompatible superparamagnetic nanoparticles, a strategy previously shown to preserve cell viability, proliferative capacity, and multicellular organization (43, 44). The experimental configuration of the magnetic force tensiometer is illustrated in Fig. 1*A*. Spheroids formed from magnetically labeled cells in 1 mm agarose microwells were matured for 2 d before being transferred into a temperature-controlled chamber positioned above a cylindrical permanent magnet generating a uniform magnetic field gradient. The millimetric size is required for capillary effects to dominate over tissue elasticity (45), while the short 2-d maturation time limits the development of necrotic regions that could affect the measurement. Under these conditions, a volumetric magnetic body force induces controlled axisymmetric flattening while maintaining nonwetting boundary conditions at the substrate interface. At equilibrium, the spheroid shape results from the balance between magnetic stresses and effective interfacial tension, analogous to the deformation of a sessile droplet under gravity. Tumor spheroids therefore behave as cohesive active materials characterized by an emergent surface tension arising from collective cell–cell adhesion, cortical tension, and higher-order tissue organization.

Following imaging of spheroid deformation under magnetic forcing, the magnetic moment of each spheroid was quantified using a benchtop magnetometer previously validated against standard magnetometry approaches. This rapid (less than 1 min) measurement provides the volumetric magnetization $M_{v}$ (magnetic moment per unit volume), which directly defines the capillary constant

[1] $$c\;=\;\frac{M_{v}\nabla \;B}{\gamma },$$

where ∇B denotes the magnetic field gradient and γ the effective surface tension of the multicellular spheroid. Under these conditions, the equilibrium spheroid profile obeys the axisymmetric Laplace equation

[2] $$\frac{d\varphi }{\mathrm{ds}}\;=\;2b\;+\;cz\;-\;\frac{\sin(\varphi )}{x},$$

where φ is the local tangent angle along the interface, s the curvilinear coordinate, and b the apex curvature.

Experimental spheroid profiles were fitted to Eq. **2** using a dedicated MATLAB routine to extract [parameters], from which the effective surface tension was determined. The complete workflow is summarized in Fig. 1*B* for breast cancer CAF-containing spheroids. The approach was applied across 19 experimental conditions, including 8 tumor cell lines, 3 CAF populations, and 8 heterotypic 70:30 tumor–CAF mixtures. Representative deformation images, fitted profiles, and surface tension values are shown in Fig. 1*C*. More than 350 independent measurements were performed, with at least 8 spheroids analyzed per condition. All individual measurements are shown in Fig. 1*D*, with mean values reported in *SI Appendix*, Table S1. Across the panel, effective surface tension spans nearly four orders of magnitude. Within breast cancer models, MDA-MB-231 displays the lowest surface tension (fractions of mN/m), MCF7 and T47D intermediate values (a few mN/m), and Hs578T the highest values (up to several tens of mN/m). A similar hierarchy is observed in pancreatic models (MiaPaCa < PANC-1 < hPAF), while A375 spheroids exhibit low surface tension comparable to MDA-MB-231 and MiaPaCa. In contrast, all CAF populations consistently exhibit high surface tension, typically ranging from 20 to 100 mN/m.

### Tumor Spheroid Morphology Scales with Effective Surface Tension Across Cancer and Stromal States.

**Fig. 2.**
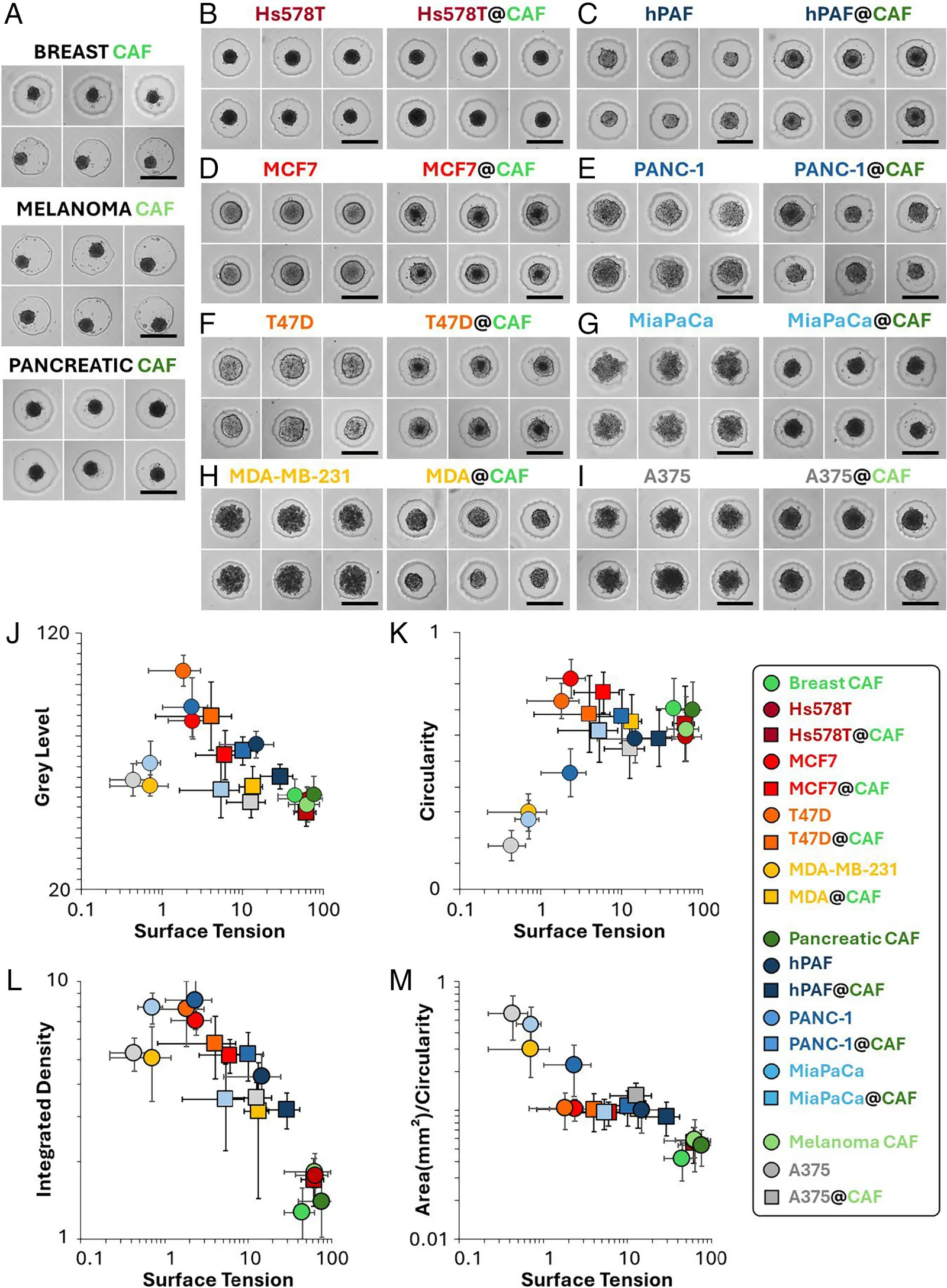
Surface tension governs multicellular spheroid morphogenesis across tumor and stromal states. (*A*–*I*) Representative brightfield images of spheroids formed in 500 µm agarose microwells 2 d after seeding 2,000 cells per microwell. (*A*) CAF spheroids formed from breast CAFs, pancreatic CAFs, and melanoma CAFs. (*B*–*I*) Tumor spheroids formed in the absence or presence of their corresponding CAFs: Hs578T (*B*), MCF7 (*C*), T47D (*D*), MDA-MB-231 (*E*), hPAF (*F*), PANC-1 (*G*), MiaPaCa (*H*), and A375 (*I*). (Scale bar, 500 µm.) (*J*–*M*) Quantitative morphological analysis of spheroids plotted as a function of effective surface tension measured in Fig. 1. (*J*) Mean gray level of spheroids. (*K*) Circularity. (*L*) Integrated density. (*M*) Ratio between projected spheroid area and circularity. Each point corresponds to the mean value obtained from approximately 200 independent spheroids for each of the 19 experimental conditions, collected across two independent experiments. The full dataset is provided in Source Data file.

To investigate whether effective surface tension controls spheroid morphogenesis, spheroids were generated in miniaturized agarose microwells embedded in 6-well plates. These 500-µm microwells, smaller than those used for magnetic tensiometry (1 mm), yielded spheroids of ~300 µm, closer to physiological size and enabling parallel formation and imaging under standardized conditions. A total of 2,000 cells were seeded per microwell, and spheroid morphology was analyzed after 2 d of maturation. Representative brightfield images are shown in Fig. 2 *A*–*I* for the three CAF populations alone and for the eight tumor cell lines with or without corresponding CAFs (70:30 cancer:CAF ratio). Clear differences in spheroid compactness, optical density, boundary smoothness, and overall morphology were observed across conditions. Additional representative images are provided in *SI Appendix*, Figs. S1–S4.

Quantitative image analysis was then used to extract morphological descriptors, which were plotted as a function of effective surface tension (Fig. 2 *J*–*M*). Mean gray level (Fig. 2*J*), reflecting light absorption and optical compactness, revealed distinct scaling behaviors for epithelial and mesenchymal states. In both groups, increasing surface tension was associated with darker, more compact spheroids. However, mesenchymal spheroids remained more absorbing across the full surface tension range, suggesting greater internal optical heterogeneity and less homogeneous organization. Circularity showed a similar trend (Fig. 2*K*): spheroids became progressively more circular with increasing surface tension, consistent with stronger effective interfacial cohesion. Epithelial MCF7 and T47D spheroids nevertheless displayed higher circularity than mesenchymal counterparts at intermediate surface tensions, indicating that epithelial organization also contributes to shape regularity.

Two further scaling relationships emerged from integrated density (Fig. 2*L*) and the ratio of projected area to circularity (Fig. 2*M*). Integrated density, reflecting both optical compactness and internal heterogeneity, decreased with increasing surface tension, indicating progressively more homogeneous and cohesive organization. Likewise, the area-to-circularity ratio decreased continuously by more than one order of magnitude with increasing surface tension. Together, these relationships establish a quantitative link between effective surface tension and collective tumor architecture.

### Surface Tension and CAFs Jointly Regulate Chemotherapeutic Resistance in Tumor Spheroids.

**Fig. 3.**
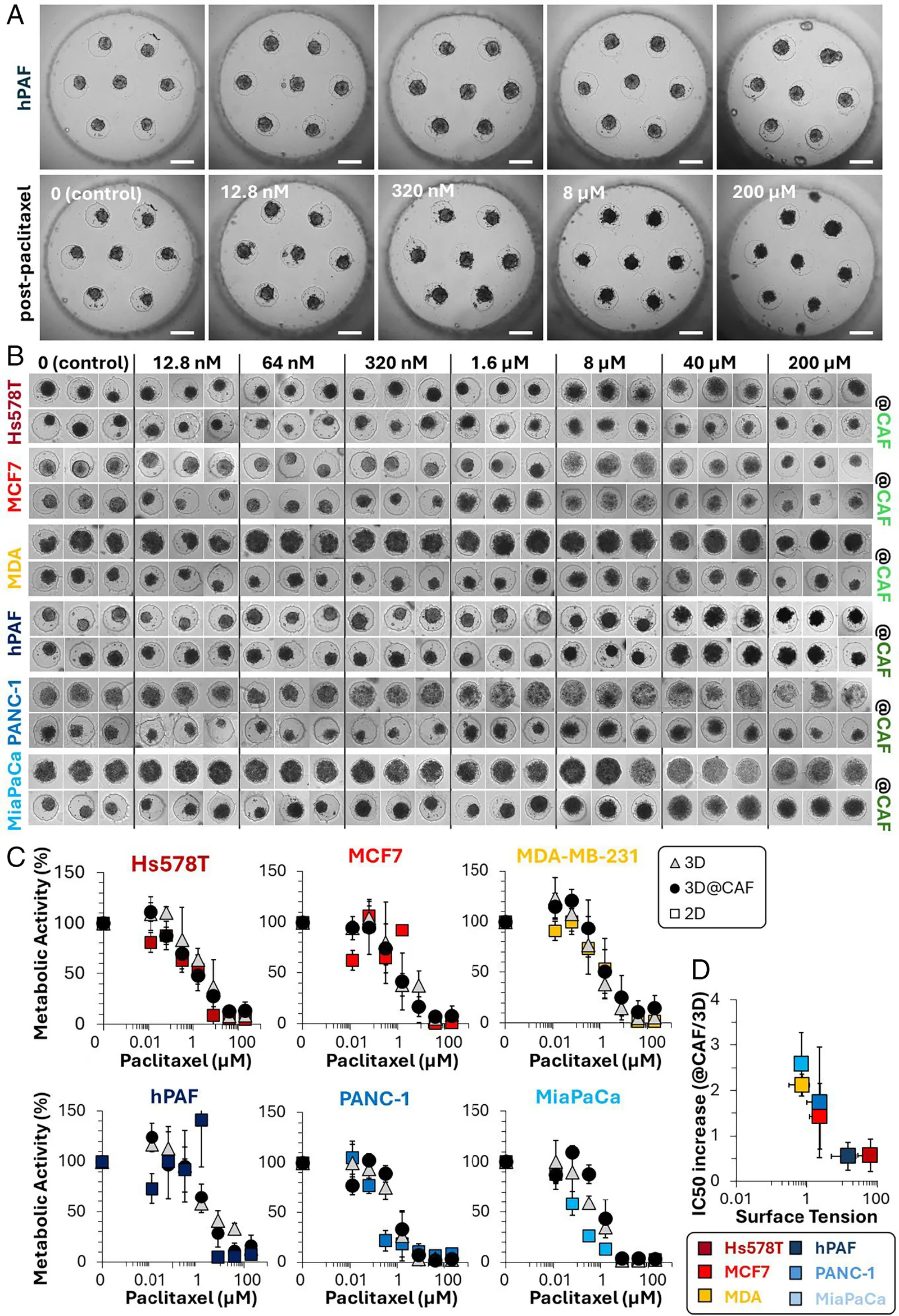
Interfacial mechanics and stromal composition modulate multicellular resistance to paclitaxel. (*A*) Representative brightfield images of hPAF spheroids in 96-well-compatible agarose microwell arrays before and after 2 d of paclitaxel exposure, showing untreated controls and increasing concentrations from 12.8 nM to 200 µM. (Scale bar, 500 µm.) (*B*) Representative endpoint images after 2 d of paclitaxel treatment for Hs578T, MCF7, MDA-MB-231, hPAF, PANC-1, and MiaPaCa spheroids cultured alone or with corresponding CAFs. Three representative spheroids are shown per condition across untreated and treated conditions. Additional images are provided in *SI Appendix*, Fig. S5. (Scale bar, 500 µm.) (*C*) Paclitaxel dose–response curves based on metabolic activity after 2 d of treatment in 2D cultures, homotypic 3D spheroids, and heterotypic tumor–CAF spheroids. IC50 values were obtained from nonlinear fits. Values represent means from three independent experiments, with six wells per condition and seven spheroids per well. Full datasets are provided in *SI Appendix*, Figs. S6–S8. (*D*) Ratio of IC50 values in heterotypic vs. cancer-only spheroids plotted against effective surface tension. CAF-mediated chemoresistance is strongest at low surface tension and decreases with increasing tissue cohesion.

Paclitaxel was used to investigate how effective surface tension and CAF incorporation influence chemotherapeutic response in breast cancer (Hs578T, MCF7, MDA-MB-231 with breast CAFs) and pancreatic (hPAF, PANC-1, MiaPaCa with pancreatic CAFs) models. To enable controlled drug screening at the spheroid scale, agarose micropatterning was miniaturized to a 96-well-compatible format, with seven independent spheroids per drug concentration. Representative hPAF spheroids exposed for 2 d to increasing paclitaxel concentrations (12.8 nM to 200 µM) are shown in Fig. 3*A*. Treatment induced progressive darkening and partial structural dissociation, consistent with alterations in multicellular organization and viability. Representative endpoint images for all tumor systems are shown in Fig. 3*B* for tumor-only and heterotypic cancer–CAF spheroids (70:30), with additional images in *SI Appendix*, Fig. S5. IC50 values were determined from metabolic activity after 2 d of treatment, using six technical replicates across three independent biological repeats. IC50 values were also measured in conventional 2D cultures for all six tumor cell lines. Individual measurements and dose–response fits are provided in *SI Appendix*, Figs. S6–S8, with corresponding values summarized in *SI Appendix*, Table S4. Fig. 3*C* compares mean IC50 values across 2D cultures, homotypic 3D spheroids, and heterotypic cancer–CAF spheroids. Transition from 2D to 3D generally increased chemoresistance, although the magnitude varied across cell types. MCF7, MDA-MB-231, and hPAF showed limited increases, whereas Hs578T, PANC-1, and MiaPaCa exhibited increases of up to approximately fourfold.

These three cases correspond to distinct biological scenarios. In Hs578T spheroids, the increased resistance may reflect their high effective surface tension and strong cohesive organization, potentially limiting drug penetration. In contrast, PANC-1 and MiaPaCa exhibit low surface tension but high intrinsic sensitivity in 2D, with substantial resistance emerging upon spheroid formation. hPAF, which combines high surface tension with high intrinsic 2D resistance, showed only a marginal increase in IC50 (1.1-fold), consistent with saturation of chemoresistance at the single-cell level. CAF populations similarly showed an approximately twofold increase in IC50 upon transition from 2D to 3D, consistent with their high surface tension regime.

Overall, multicellular cohesion in the 20 to 100 mN/m range was associated with increased chemotherapeutic resistance in spheroids, unless cells were intrinsically highly resistant. Comparison of heterotypic cancer–CAF and cancer-only spheroids (Fig. 3*D*) further revealed a strong dependence on the surface tension regime. In low surface tension cancer models, CAF incorporation increased IC50, whereas in high surface tension systems (Hs578T and hPAF), CAF addition instead reduced IC50. Thus, CAFs modulate chemotherapeutic response in a surface tension-dependent manner, linking stromal composition, multicellular cohesion, and drug sensitivity.

### CAF Spatial Organization in Heterotypic Cancer-CAF Spheroids Deviates from Surface Tension Predictions.

**Fig. 4.**
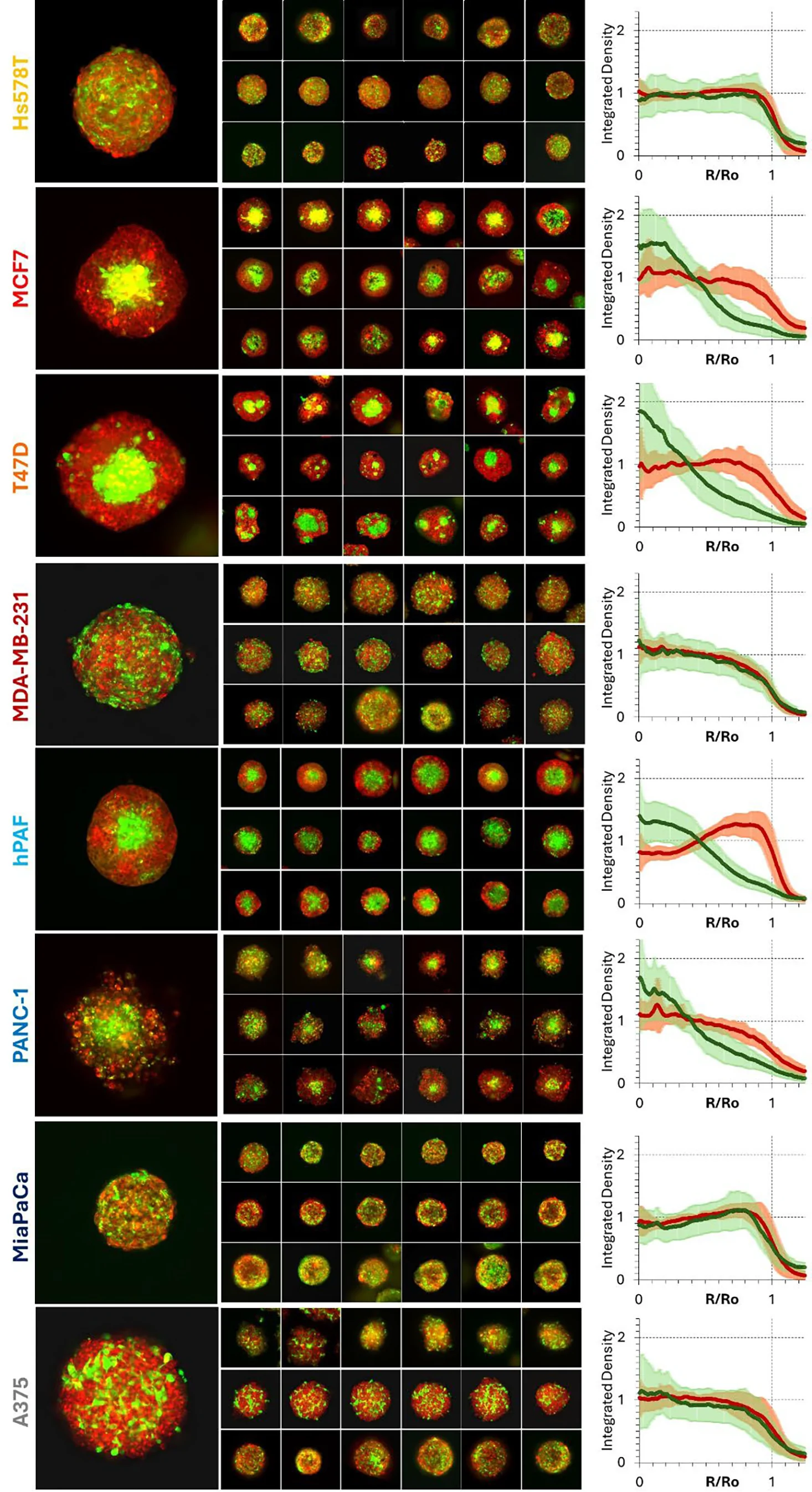
Spatial organization of CAFs within heterotypic cancer spheroids. Fluorescence imaging of cancer cells (PKH26, red) and CAFs (PKH67, green) in heterotypic spheroids formed at a 70:30 ratio. From *Top* to *Bottom*, rows correspond to breast cancer cell lines (Hs578T, MCF7, T47D, MDA-MB-231), pancreatic cancer cell lines (hPAF, PANC-1, MiaPaCa), and melanoma (A375). For each condition, representative spheroid and fluorescence images are shown (18 independent spheroids per condition). *Right* panels show mean normalized radial fluorescence profiles (n = 18) as a function of normalized radial position (R/R_o_), with R/R_o_ = 0 corresponding to the spheroid center and 1 to the periphery. Field of view, 500 × 500 µm. Individual measurements are provided in Source Data file (sheet “Fig. 4”).

Given their high effective surface tension, CAFs would be expected to accumulate at the spheroid core, in contrast to the commonly assumed peripheral organization. Only in Hs578T spheroids, where CAF and tumor cell surface tensions are comparable, would a fully intermixed organization be expected. To test these predictions, cancer cells and CAFs were fluorescently labeled red and green, respectively, and mixed at a 70:30 ratio before spheroid formation across the eight cancer conditions. Fig. 4 shows representative fluorescence images and averaged radial intensity profiles. Cell distribution was quantified using the ImageJ Radial Profile plugin at a fixed optical section. Radial position was normalized to spheroid radius R/Ro, with 0 corresponding to the center and 1 to the periphery.

Consistent with the surface tension hierarchy, CAF enrichment at the core was observed in MCF7, T47D, PANC-1, and hPAF spheroids. Hs578T spheroids, with comparable cancer and CAF surface tensions, displayed the expected fully intermixed organization. By contrast, this picture broke down in MDA-MB-231, MiaPaCa, and A375 spheroids. Despite their surface tensions being nearly two orders of magnitude lower than those of CAFs, which would predict strong CAF core localization, CAFs remained homogeneously distributed and fully intermixed with cancer cells, with no detectable radial segregation (neither IN nor OUT).

Overall, radial fluorescence analysis shows that surface tension alone cannot account for CAF organization across all conditions, with several systems deviating from the expected mechanical ordering.

### Temporal Ordering of Cancer Spheroid Formation and CAF Recruitment Reshapes (or Not) CAF-Cancer Spatial Organization.

**Fig. 5.**
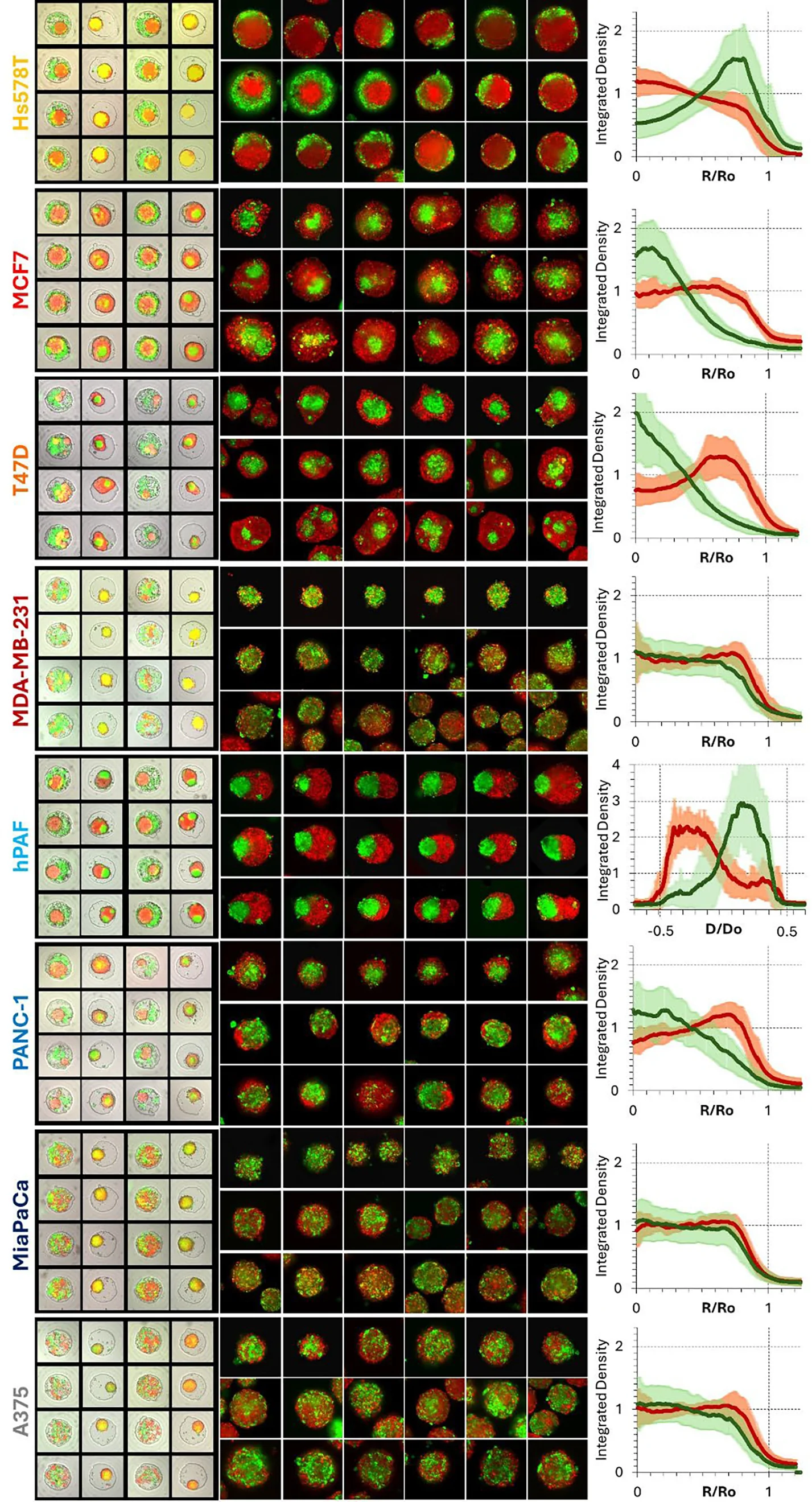
Sequential recruitment of cancer-associated fibroblasts into preformed tumor spheroids reveals distinct invasion and organization regimes. Cancer spheroids were first formed for 2 d before CAF introduction. CAFs were seeded above and below the preformed spheroids to facilitate sedimentation and interaction within agarose microwells. Rows correspond to breast cancer cell lines (Hs578T, MCF7, T47D, MDA-MB-231), pancreatic cancer cell lines (hPAF, PANC-1, MiaPaCa), and melanoma (A375). *Left* panels show bright-field and fluorescence images before CAF addition and after 2 d of coculture, with three representative images per condition. *Middle* panels show fluorescence images of cancer cells (PKH67, green) and CAFs (PKH26, red). *Right* panels show mean radial organization for radially symmetric spheroids; hPAF dumbbell-like structures were quantified using directional distance measurements. The full dataset is provided in Source Data file (sheet “Fig. 5”).

The results above indicate that neither the surface tension hierarchy, which predicts CAF “IN” localization, nor the prevailing paradigm of CAF “OUT” organization is consistently observed. We therefore asked whether the mode of spheroid formation could influence CAF organization. In the initial experiments, cancer cells and CAFs were mixed from the outset, effectively embedding stromal cells within the developing tumor tissue. To mimic a more sequential recruitment process, cancer spheroids were first allowed to form for 2 d before CAF addition (Fig. 5). CAFs were initially observed as individual cells surrounding the preformed cancer spheroids and, after two additional days, became incorporated to different extents. Fig. 5 shows the temporal sequence together with fluorescence images and spatial organization analyses.

Remarkably, Hs578T spheroids now developed a clear CAF shell surrounding the preformed cancer spheroid, demonstrating that the peripheral organization observed in tumors can emerge under sequential recruitment. MCF7 and T47D behaved differently: CAFs invaded the preformed spheroids and progressively accumulated at the center, forming a well-defined CAF-rich core. A similar, although weaker, trend was observed for PANC-1. Thus, the high CAF surface tension was sufficient to drive CAF penetration into preformed spheroids and reposition the core toward a CAF-dominated configuration.

For the looser MDA-MB-231, MiaPaCa, and A375 spheroids, easier CAF penetration and central accumulation might be expected. Instead, after 2 d of coculture, CAFs remained fully intermixed with cancer cells, reproducing the mixed architecture observed after simultaneous coseeding. hPAF spheroids revealed yet another behavior: CAFs did not invade the preformed spheroid but formed an attached dumbbell-like structure, consistent with the higher surface tension of hPAF compared with MCF7, T47D, and PANC-1. Because radial analysis was not applicable to this geometry, spatial partitioning was quantified using a longitudinal distance metric (D/D_o_). Applied also to T47D spheroids with radial symmetry, this metric correlated well with radial measurements (*SI Appendix*, Fig. S9).

Overall, temporal separation between cancer assembly and CAF recruitment revealed distinct organizational outcomes, including peripheral CAF shells in Hs578T, CAF-rich cores in MCF7 and T47D, and segregated dumbbell-like structures in hPAF. In contrast, lower-surface-tension MDA-MB-231, MiaPaCa, and A375 spheroids remained intermixed. These results highlight the ability of CAFs to penetrate preformed tumor spheroids and show that their final organization depends strongly on both tissue mechanics and assembly history.

### A Unified OUT–IN Framework Reveals EMT-Dependent CAF Organization Beyond Surface Tension.

**Fig. 6.**
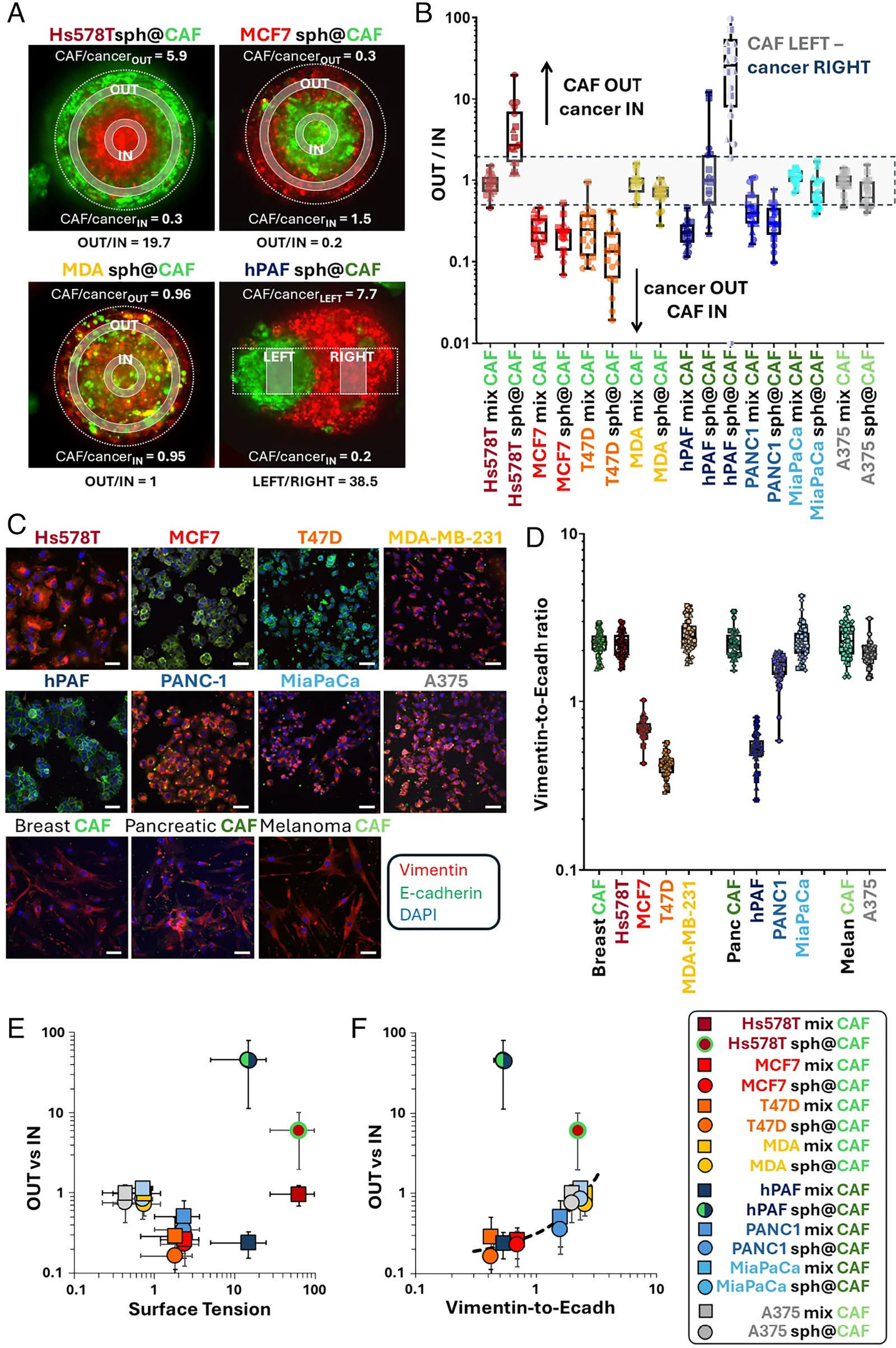
CAF spatial organization is governed by epithelial–mesenchymal state rather than surface tension alone. (*A*) Definition of the OUT/IN parameter used to quantify CAF localization from radial fluorescence profiles. IN and OUT correspond to fluorescence averaged over R/R_o_ = 0.2 to 0.3 and 0.7 to 0.8, respectively. OUT/IN > 2 indicates peripheral CAF localization, ≈1 homogeneous mixing, and <0.5 central accumulation. For nonradial dumbbell-like structures, a LEFT/RIGHT metric based on D/D_o_ was used. (*B*) CAF organization across the 16 heterotypic conditions from Figs. 4 and 5. Coseeding and sequential assembly are denoted cancer mix CAF and cancer sph@CAF, respectively. Epithelial and mesenchymal states show strongly different organization, while assembly history has a major effect only in Hs578T and hPAF. (*C*) Representative Vimentin and E-cadherin immunofluorescence images for all cancer cell lines and CAF populations. (Scale bar, 100 µm.) (*D*) Quantification of Vimentin and E-cadherin fluorescence and corresponding Vimentin-to-E-cadherin ratio as an EMT metric. Epithelial and mesenchymal states differ significantly (****), with PANC-1 showing an intermediate phenotype. (*E*) OUT/IN as a function of tumor spheroid surface tension. No clear relationship is observed. (*F*) OUT/IN as a function of the Vimentin-to-E-cadherin ratio. Increasing EMT is associated with a shift from central CAF accumulation toward homogeneous mixing, with Hs578Tsph@CAF and hPAFsph@CAF as exceptions. Statistical analyses were performed on log10-transformed values using one-way ANOVA with multiple comparisons; relevant comparisons are reported in *SI Appendix*, Tables S5 and S6 and complete outputs in the Source Data. Each point represents the mean of three independent experiments.

Fig. 6 was designed to identify the organizing principle underlying the CAF distributions observed in Figs. 4 and 5. Fig. 6*A* introduces a quantitative OUT–IN parameter derived from normalized radial fluorescence profiles, based on CAF and cancer cell intensities in two radial windows: R/R_o_ = 0.2 to 0.3 (IN), and 0.7 to 0.8 (OUT). OUT–IN values above 2 indicate peripheral CAF enrichment, values close to 1 homogeneous mixing, and values below 0.5 central accumulation. For dumbbell-like structures lacking radial symmetry, organization was quantified along a linear axis using two corresponding spatial windows (D/D_o_ = 0.2 to 0.35 and 0.65 to 0.8). Fig. 6*B* reports OUT–IN values for all 16 heterotypic conditions from Figs. 4 and 5, comparing coseeding (“cancer mix CAF”) and sequential assembly (“cancer sph@CAF”).

MDA-MB-231, MiaPaCa, and A375 systems remained close to 1 under both assembly conditions, indicating homogeneous mixing despite their very low tumor surface tension. MCF7 and T47D consistently showed values below 0.5, indicating strong central CAF accumulation, while PANC-1 displayed weaker inward enrichment. Only hPAF and Hs578T showed a strong dependence on assembly history. hPAFmixCAF spheroids exhibited central CAF accumulation, whereas hPAFsph@CAF formed dumbbell-like structures with adjacent CAF-rich and tumor-rich domains. Hs578TmixCAF remained intermixed, while Hs578Tsph@CAF developed a clear core-shell organization with OUT–IN > 2. Overall, OUT–IN values did not correlate with surface tension (Fig. 6*E*), confirming that surface tension alone cannot explain CAF organization.

The EMT status of the different models was then considered. MDA-MB-231, MiaPaCa, A375, and Hs578T were classified as mesenchymal-like, MCF7 and T47D as epithelial, and PANC-1 as intermediate. EMT status was confirmed by E-cadherin and Vimentin staining (Fig. 6 *C* and *D*), and an averaged Vimentin-to-E-cadherin ratio was used as an EMT metric. Strikingly, OUT–IN values correlated with this metric (Fig. 6*F*): epithelial cells favored central CAF accumulation, whereas mesenchymal cells converged toward homogeneous mixing. This relationship held across conditions, with only two exceptions corresponding to sequential assembly in Hs578T and hPAF.

Overall, CAF organization is not determined by surface tension alone. When cancer cells and CAFs are coseeded, epithelial states favor central CAF accumulation whereas mesenchymal states favor mixing. Peripheral organization emerges only when CAFs are recruited after tumor spheroid formation and, among the models tested, only in high-surface-tension systems.

## Discussion

Beyond its mechanistic implications for multicellular organization, this work challenges a common assumption in tumor biology: that CAFs spontaneously form a stable peripheral stromal shell around tumor tissues. Such a configuration is often invoked in models of tumor progression, mechanical confinement, immune exclusion, and therapy resistance. However, across the tumor types and assembly conditions investigated here, peripheral organization is not a general outcome. Instead, CAFs display heterogeneous spatial behaviors, ranging from complete intermixing to central accumulation and, in only two conditions, clear peripheral organization.

Although surface tension is the main physical parameter considered here, other mechanical properties, including nonlinear tissue elasticity (46), may also contribute to multicellular organization. Surface tension, however, plays an important role in cell segregation. Originating from Steinberg’s differential adhesion hypothesis (20, 21, 24), including the role of interfacial tension between two cell populations (47), and extended to multicellular physics (25, 26, 36), the surface tension framework predicts segregation according to effective cohesion. Accordingly, CAFs, which occupy the high end of the surface tension hierarchy, would be expected to accumulate at the core of cancer spheroids. This prediction is recovered in epithelial tumor cell types but fails in mesenchymal-like models such as MDA-MB-231, MiaPaCa, and A375, where CAFs remain dispersed despite strong surface tension contrasts. Thus, tumor–stroma organization cannot be reduced to passive minimization of interfacial energy, but must also incorporate active processes such as motility, invasion, and phenotype-dependent remodeling. This is consistent with the established role of EMT in regulating adhesion, motility, and collective invasion (48–50), highlighting the coupling between tumor mechanics and cell state.

A key outcome of this study is the robust correlation between CAF organization and epithelial-to-mesenchymal state, quantified by the Vimentin-to-E-cadherin ratio. Epithelial-like spheroids consistently favor central CAF accumulation, even when CAFs are introduced after tumor preassembly, whereas mesenchymal systems favor persistent mixing. Only under a restricted combination of high tumor surface tension and sequential stromal recruitment does peripheral organization emerge. In Hs578T, this produces a clear CAF shell, whereas hPAF forms two adjacent CAF-rich and tumor-rich domains in a dumbbell-like structure. Thus, CAF organization cannot be understood from static mechanical or phenotypic parameters alone, but also depends on how the tissue is assembled, suggesting a form of “structural memory” in tumor architecture.

Taken together, these findings indicate that CAF organization is not governed by a universal self-organization principle, but by the interplay between tissue cohesion, EMT state, and stromal recruitment history. This introduces a strong kinetic and historical component into tumor architecture, consistent with nonequilibrium tissue physics, where final structure depends on assembly pathway rather than equilibrium state alone (25, 51) and where dynamical modeling becomes necessary (52). Differential proliferation between cancer cells and CAFs should also influence the final architecture (53), and is particularly relevant in the sequential assembly experiments here, where the 2-d period of cancer cell proliferation before CAF addition should be considered. Heterotypic adhesion forces may also contribute, as recent work shows that adhesion between cancer cells and macrophages influences early tumor development (31).

Purely physical description breaks down in mesenchymal-like cancer cell lines, where CAFs and cancer cells remain mixed despite large differences in surface tension. In these systems, passive sorting appears to be counterbalanced by active cellular processes, including intercalation and local remodeling. Mesenchymal states are characterized by low E-cadherin, high vimentin, and enhanced plasticity, motility, and invasion (48, 54–56). Persistent motility and matrix remodeling may reduce the effective energetic cost of heterotypic contacts (48, 54, 57, 58), suggesting that interfacial tension between epithelial and mesenchymal populations should be considered in addition to bulk tissue cohesion (59). This interpretation is consistent with active matter descriptions of tumors as systems that can transition from jammed, solid-like states to more fluid-like regimes, in which organization reflects the balance between adhesion, motility, persistence, and intercalation rather than passive energy minimization (58, 60–63).

From a translational perspective, this framework could help reconcile apparently conflicting observations in patient samples and experimental tumor models. CAFs have been reported at invasive fronts (14), in peritumoral stromal rims (64), within intratumoral niches (1), or diffusely distributed depending on tumor type and stage. Importantly, peripheral CAF organization is observed in lung cancer (65, 66). The spheroid models used here do not reproduce the full complexity of tumor tissues, where CAFs are typically recruited progressively rather than coassembled with cancer cells from the outset. This temporal recruitment favors peripheral organization, as observed in the sequential assembly experiments here. Most importantly, extracellular matrix deposition and ECM–cell interactions, particularly involving matrix produced by CAFs themselves, can also strongly influence tissue architecture (67) and will need to be considered next. Nevertheless, these results may already provide a physical basis for understanding how peripheral CAF organization can emerge as a context- and history-dependent state in tumors.

To conclude, this work revisits the assumption that CAFs form a predictable peripheral barrier in tumors. CAFs occupy the high end of the surface tension hierarchy, which would predict central accumulation rather than peripheral localization. Yet surface tension alone cannot account for their spatial organization: mesenchymal cancer cells with much lower surface tension can remain fully intermixed with CAFs, whereas epithelial cells favor CAF accumulation at the center. Peripheral organization emerges only under specific conditions combining high tumor surface tension with sequential CAF recruitment. CAF position therefore reflects a conditional interplay between surface tension, tumor identity, and assembly history rather than a universal stromal architecture.

## Materials and Methods

Detailed materials methods are provided in *SI Appendix*.

### Cell Lines and Cancer-Associated Fibroblast Culture Conditions.

Human breast (Hs578T, MCF7, T47D, MDA-MB-231), pancreatic (HPAF-II, PANC-1, MiaPaCa-2), and melanoma (A375) cancer cells were obtained from ATCC, and breast, pancreatic, and melanoma CAFs from Vitro Biopharma.

### Magnetic Force Tensiometry.

Magnetically labeled cells were seeded into 1 mm agarose microwells and spheroids were matured for 2 d before deformation under a 25 T‧m^–1^ magnetic field gradient. Spheroid profiles were fitted to the analytical deformation model, and effective surface tension was determined from the fitted parameters and independent volumetric magnetization measurements using a benchtop magnetic sensor (68).

### Spheroid Formation in 6-Well Agarose Microwells.

Spheroids were generated in 2% agarose microwells made from custom 3D-printed molds (69). Cells were seeded at 2,000 cells per 500 µm microwell and cultured for 2 d. Heterotypic spheroids were generated at a 70:30 cancer-to-CAF ratio; sequential assembly involved addition of CAFs to preformed cancer spheroids.

### Spheroid Formation in 96-Well Plates for Drug-Response Assay.

Spheroids were generated in 96-well agarose arrays containing seven 500 µm microwells per well and matured for 2 d before paclitaxel treatment. Cancer-only and 70:30 cancer–CAF spheroids were exposed to paclitaxel for 48 h, and metabolic activity was measured by Alamar Blue for IC50 determination; parallel 2D cultures were analyzed under the same conditions.

### Spheroid Imaging and Analysis of CAF Spatial Localization.

Fluorescently labeled spheroids were fixed, optically cleared using glycerol–fructose–urea, and imaged by spinning-disk confocal microscopy. Radial fluorescence profiles were extracted from sections located 100 ± 25 µm above the spheroid bottom using the Fiji Radial Profile plugin.

### Immunofluorescence Staining and Confocal Imaging of EMT Markers.

Cancer cell lines and CAFs were fixed, permeabilized, and stained for E-cadherin and vimentin with fluorescent secondary antibodies and DAPI. Images were acquired by spinning-disk confocal microscopy using a 10× objective.

### Statistical Analysis.

Statistical analyses were performed using GraphPad Prism. Surface tension and fluorescence values were log10-transformed and compared using one-way ANOVA with multiple-comparison tests; full pairwise comparisons were computed. Significance was defined as ns (*P* ≥ 0.05), *(*P* < 0.05), **(*P* < 0.01), ***(*P* < 0.001), and ****(*P* < 0.0001).

## Supplementary Material

Appendix 01 (PDF)

Dataset S01 (XLSX)

## Data Availability

Study data are included in the article and/or supporting information.
