# Supplementary material for: Cell identity competes with surface tension to determine stromal organization in tumor spheroids beyond the CAF shell paradigm
Source: Proc Natl Acad Sci U S A. 2026 Oct 12;123(42):e2624455123. doi: 10.1073/pnas.2624455123 (PMC13684131; doi:10.1073/pnas.2624455123)
Supplement: Supplementary file 1 [file pnas.2624455123.sapp.pdf]

Updated August 2025
PNAS

Supplementary Informations for

Cell identity competes with surface tension to determine stromal organization in tumor spheroids beyond the CAF shell paradigm

Claire Wilhelm, Physics of Cells and Cancer, PCC, CNRS UMR168, Institut Curie, Sorbonne Université, PSL Research University, 75005 Paris, France; **Email:** claire.wilhelm@cnrs.fr

This PDF file includes: Supplementary Methods; Tables S1 to S7; Figs. S1 to S11;

Supplementary Materials and Methods

Cell lines and cancer-associated fibroblast culture conditions

Human breast cancer cell lines Hs578T, MCF7, T47D, and MDA-MB-231, pancreatic cancer cell lines HPAF-II, PANC-1, and MiaPaCa-2, and the melanoma cell line A375 were maintained under standard humidified conditions at 37 °C and 5% CO_2. All cancer cell lines were obtained from the American Type Culture Collection (ATCC, Manassas, VA, USA) and used according to the supplier's specifications. Breast cancer cell lines MDA-MB-231 (ATCC-HTB-26), MCF7 (ATCC-HTB-22), T47D (ATCC-HTB-133), and Hs578T (ATCC-HTB-126), pancreatic cancer cell lines PANC-1 (ATCC-CRL-1469), MiaPaCa-2 (ATCC-CRL-1420), and HPAF-II (ATCC-CRL-1997), and melanoma cell line A375 (ATCC-CRL-1619) were used. Cell line identity was confirmed by short tandem repeat (STR) profiling and matched against reference profiles. Detailed STR profiles are reported in Supplementary Table S1. Cells were used within a maximum of 10 passages after thawing to minimize phenotypic drift. Hs578T, MCF7, MDA-MB-231, PANC-1, MiaPaCa-2, and A375 were cultured in Dulbecco's Modified Eagle Medium (DMEM, high glucose) supplemented with 10% fetal bovine serum (FBS), 2 mM L-glutamine, and 1% penicillin–streptomycin. T47D cells were cultured in RPMI-1640 medium supplemented with 10% FBS, 2 mM L-glutamine, 1% penicillin–streptomycin, and recombinant human insulin (10 $\mu g \cdot mL^{-1}$). HPAF-II cells were cultured in Eagle's Minimum Essential Medium (EMEM) supplemented with 10% FBS and 1% penicillin–streptomycin. Cancer-associated fibroblasts (CAFs) derived from breast, pancreatic, and melanoma tumors were obtained from Vitro Biopharma (USA). Breast CAFs (CAF06), pancreatic CAFs (CAF08), and melanoma CAFs (CAF09) were used according to supplier specifications. CAFs were provided cryopreserved at low passage number and expanded following manufacturer-recommended protocols. Breast and melanoma CAFs were cultured in VitroPlusIII Low-Serum Complete Medium (Vitro Biopharma, Cat. No. PC00B1), while pancreatic CAFs were maintained in MSC-GRO™ Pancreatic Stellate CAF Maintenance Medium (Vitro Biopharma, Cat. No. PC00B5). CAFs were used at passage ≤5 for all experiments. All cells were maintained at 37 °C in a humidified atmosphere containing 5%

CO_2 and routinely passaged using trypsin–EDTA prior to confluence. All cell lines and CAF populations were routinely tested and confirmed negative for mycoplasma contamination.

Microwell fabrication

All spheroids used in this study were generated using custom-designed, in-house fabricated 3D-printed molds. Micropillar arrays were produced using a DigitalWax 028J Plus 3D printer (DWS Systems) and printed with LOCTITE 3D MED412 resin, certified biocompatible according to ISO 10993-5 and ISO 10993-10 standards. The spatial resolution of the printing process is 25 × 25 µm. Three distinct mold geometries were designed to match experimental requirements and to ensure reproducible spheroid formation across all assays. For 24-well plates, molds were designed to generate a single large central microwell per well, defined by a cylindrical pillar of 1 mm diameter, used primarily for the magnetic force tensiometry experiments (Figure 1). For 6-well plates, high-density arrays of 401 microwells (500 µm diameter, 500 µm depth) were fabricated per well, enabling large-scale spheroid production for morphological analyses (Figures 2, 4, and 5). For 96-well plates, molds containing seven microwells per well (500 µm diameter and 500 µm height) were used for high-throughput drug response experiments (Figure 3). All designs feature a conical top on the pillars, acting as a funnel to facilitate cell capture. All three designs, together with their geometrical specifications, are shown in Supplementary Figure S11. Microwells were then fabricated by casting molten 2% (w/v) agarose in PBS into each well, followed by placement of the 3D-printed stamps. After gelation at room temperature for 15 min, the stamps were carefully removed, leaving well-defined non-adhesive microwell structures. Agarose volumes were adjusted according to plate format (200 µL for 96-well plates, 1 mL for 24-well plates, and 3.5 mL for 6-well plates).

Magnetic force tensiometry

The experimental configuration of the magnetic force tensiometer is illustrated in Fig. 1A. Cells were incubated for 30 min with citrate-coated iron oxide nanoparticles at a final iron concentration of 1 mM to enable efficient intracellular uptake prior to spheroid formation. This labeling protocol has previously been shown not to affect cell viability, proliferation, or differentiation. Cells were then washed and seeded into custom 1 mm agarose microwells (10^5 cells per well), fabricated by 3D printing, to initiate spheroid assembly. For magnetic molding, a 24-well plate equipped with an array of 3D-printed holders positioning 24 permanent magnets directly beneath each microwell was used. A magnetic pulse (1 min) was applied immediately after seeding to promote initial compaction, after which spheroids were left to self-assemble for 2 days under no external forcing. Fully formed spheroids were then transferred into a temperature-controlled chamber and positioned above a cylindrical permanent magnet generating a uniform magnetic field gradient of $25\ \mathrm{T \cdot m^{-1}}$. Under these conditions, a constant volumetric magnetic body force is applied to the tissue, inducing a controlled axisymmetric deformation while maintaining non-wetting boundary conditions at the substrate interface. At equilibrium, spheroids adopt a shape resulting from the balance between bulk magnetic stresses and interfacial tension, consistent with the sessile-drop analogy under a uniform body force. Multicellular aggregates are therefore treated as cohesive fluid-like materials characterized by an emergent effective surface tension. The magnetic forcing is described by the volumetric force density $f_v = M_v \nabla B$ (eq 1). The equilibrium interface is governed by the axisymmetric Laplace formalism (Eq. 2), while the capillary constant is defined by Eq. 3. Spheroid profiles are extracted from bright-field images and fitted to the analytical solution of the deformation model using a dedicated MATLAB-based routine. Combined with

independent measurements of volumetric magnetization, these parameters yield the effective surface tension for each multicellular aggregate. Volumetric magnetization M_v is determined using a compact benchtop magnetic sensor (Magnisense SA), enabling rapid, non-destructive quantification of spheroid magnetic moment. The measurement is based on the nonlinear superparamagnetic response of intracellular iron oxide nanoparticles. Spheroids (in 50 µL medium in 0.5 mL tubes) are subjected to dual alternating magnetic fields (f_1 = 100 kHz, 10 Oe; f_2 = 100 Hz, 200 Oe), and the detected signal corresponds to the third derivative of the magnetization at zero field, proportional to nanoparticle content. Each measurement takes approximately 30 seconds to 1 minute. Calibration against vibrating sample magnetometry (VSM) enables conversion of the sensor signal into magnetic moment. As previously established, a linear correlation is maintained across a broad range of nanoparticle concentrations. The magnetic moment is normalized by spheroid volume to obtain M_v. This pipeline is applied across all 19 experimental conditions, including cancer cell lines, CAF populations, and heterotypic 50:50 tumor–CAF mixtures, corresponding to more than 350 independent measurements.

Spheroid formation in 6-well agarose microwells

For spheroid formation in 6-well agarose microwell plates (500 µm microwells), cells were seeded at a density of 2,000 cells per microwell, resulting in spheroids with a final diameter of 250–350 µm, with spheroid size additionally serving as a readout of collective cohesion (Fig. 2). For assembly, cells were detached by trypsinization and deposited into the large well of the 6-well plate containing the agarose-microwell array at a total density of 800,000 cells per well in 800 µL of complete medium. For heterotypic assemblies (Figures 4 and 5), cancer cells and CAFs were mixed at defined ratios prior to seeding. A 70:30 cancer-to-CAF ratio was used (560,000 cancer cells and 240,000 CAFs per well). Cells were allowed to sediment for 30 min at 37 °C, after which the volume was adjusted to 3 mL of complete culture medium. Spheroids were then cultured for 2 days to allow structural maturation prior to analysis. This protocol was used for all experiments shown in Figure 2. For Figures 4 and 5, cancer cells and CAFs were fluorescently labeled prior to seeding using PKH membrane dyes (Sigma-Aldrich) according to the manufacturer's protocol. Cancer cells were labeled with PKH26 (red fluorescence; Ex/Em ≈ 551/567 nm), whereas CAFs were labeled with PKH67 (green fluorescence; Ex/Em ≈ 490/502 nm). Briefly, cell pellets were resuspended in Diluent C and incubated with the corresponding dye solution for 5 min at room temperature. Cells were then centrifuged and resuspended in complete medium twice to remove excess dye. Following an additional 10 min incubation in complete medium to stabilize membrane incorporation and minimize cross-labeling between populations, cells were centrifuged again, resuspended at the desired cell density, mixed, and seeded as described above. This protocol was used for the spheroids analyzed in Figure 4. By contrast, for Figure 5, the protocol was adapted to a sequential assembly approach. Cancer spheroids were first generated from PKH26-labeled cancer cells by seeding 400,000 cells per well (approximately 1,000 cells per microwell) into 6-well agarose microwell arrays, and allowed to mature for 2 days. In parallel, a second agarose microwell mold was prepared and seeded with PKH67-labeled CAFs at a density of 120,000 cells per well, followed by a 15 min sedimentation step. Approximately 100 pre-formed cancer spheroids were then transferred onto the CAF-containing microwell array under gentle orbital agitation for 30 min to ensure efficient positioning of one spheroid per microwell and to promote isotropic CAF access. A second layer of CAFs (120,000 cells per well) was subsequently added on top of the system. The co-cultures were then left to mature for an additional 2 days prior to imaging and analysis.

Spheroid formation in 96-well plates for drug-response assay

For drug-response experiments, spheroids were generated in 96-well agarose microwell plates containing seven 500 µm microwells per well. Cell seeding densities were adjusted to maintain 2,000 cells per microwell equivalent, corresponding to a total of 14,000 cells per well. Breast cancer cell lines (Hs578T, MCF7, and MDA-MB-231) and pancreatic cancer cell lines (HPAF-II, PANC-1, and MiaPaCa-2) were investigated. For heterotypic conditions, breast CAFs were combined with breast cancer cell lines and pancreatic CAFs with pancreatic cancer cell lines at a CAF:cancer ratio of 30:70. Monoculture and heterotypic spheroids were formed as described above and allowed to mature for 2 days before treatment. Paclitaxel was used to evaluate the impact of CAFs on chemotherapeutic response through IC50 determination. Eight drug concentrations were tested (0, 0.0128, 0.064, 0.32, 1.6, 8, 40, and 200 µM), with six independent wells analyzed per condition. Each 96-well plate corresponded to one cell line, including four rows of cancer-only spheroids and four rows of CAF-containing spheroids. Experiments were independently repeated three times using freshly thawed batches of both cancer cells and CAFs. Conventional 2D drug-response experiments were performed in parallel for cancer cell lines alone. Cells were seeded at 14,000 cells per well in standard culture plates 2 days before drug exposure, using identical paclitaxel concentrations and replicate numbers as for spheroid experiments. Paclitaxel exposure was maintained for 48 h prior to viability analysis using the Alamar Blue assay (Thermo Fisher Scientific). This assay relies on the reduction of resazurin into the fluorescent metabolite resorufin by metabolically active cells. A 10% Alamar Blue working solution was added to each well (200 µL final volume) and incubated for 3 h at 37 °C. Fluorescence was then measured using an EnSight multimode plate reader (PerkinElmer) with excitation/emission wavelengths of 560/590 nm. Relative metabolic activity was normalized to untreated controls for IC50 determination.

Spheroid imaging and analysis of CAF spatial localization

For spatial organization analyses (Figs. 4 and 5), spheroids were generated in 500 µm agarose microwells in 6-well plates using PKH67-labeled CAFs (green fluorescence) and PKH26-labeled cancer cells (red fluorescence), as described above. After 2 days of maturation, spheroids were collected, fixed in 4% paraformaldehyde, and subjected to an optical clearing procedure prior to fluorescence imaging. Optical clearing was performed using a glycerol–fructose–urea (GFU) clearing solution adapted from previously described refractive-index matching approaches for 3D multicellular tissues. Briefly, spheroids were sequentially incubated in increasing concentrations of GFU diluted in washing buffer (33%, 66%, then 100%) for 1 h, 1 h, and overnight, respectively. The 100% GFU solution was then renewed and spheroids were maintained in clearing solution for at least an additional 24 h prior to imaging to ensure complete optical homogenization. The final GFU clearing solution contained 50% glycerol, 2.5 M fructose, 2.5 M urea, 10.6 mM Tris buffer, and 1.1 mM EDTA in distilled water. The washing buffer consisted of PBS supplemented with 0.02% SDS, 0.1% Triton X-100, and bovine serum albumin. All clearing solutions were prepared in advance, stored at 4 °C protected from light, and used within one month. Cleared spheroids were imaged by fluorescence microscopy using a spinning-disk confocal microscope (Leica Microsystems) with identical acquisition parameters for all experimental conditions. Excitation lasers were operated at 33% power, with an exposure time of 200 ms for both fluorescence channels. PKH67-labeled CAFs were imaged using 488 nm excitation and 535–545 nm emission detection, whereas PKH26-labeled cancer cells were imaged using 535 nm excitation and 605–625 nm emission detection. Z-stack acquisitions were performed with a 5 µm step size using a 10×

objective and controlled through MetaVue acquisition software. For quantitative analysis, optical sections located at 100 ± 25 µm above the spheroid bottom were selected from each stack. Radial fluorescence intensity profiles were then extracted using the Radial Profile plugin in Fiji to quantify the spatial distribution of CAFs and cancer cells within multicellular spheroids.

Immunofluorescence staining and confocal imaging of EMT markers

The eight cancer cell lines (Hs578T, MCF7, T47D, MDA-MB-231, hPAF, PANC-1, MiaPaCa, and A375) as well as the three CAF populations (breast, pancreatic, and melanoma CAFs) were expanded in 2D culture in 96-well plates until reaching 60–80% confluence. Cells were then fixed in 4% paraformaldehyde for 1 hour at room temperature and subsequently washed thoroughly with PBS. Permeabilization was performed using 0.1% Triton X-100 in PBS for 15 minutes at room temperature, followed by blocking in 3% bovine serum albumin in PBS containing 0.1% Triton X-100 for 3 hours. Samples were incubated overnight at room temperature with primary antibodies against E-cadherin (42027; Thermo Fisher Scientific; 1:200) and vimentin (GP53; Progen Biotechnik GmbH; 1:400), diluted in blocking buffer. After several washes with PBS, cells were incubated overnight with Alexa Fluor 488-conjugated goat anti-mouse IgG (A11029; Thermo Fisher Scientific) and Alexa Fluor 647-conjugated goat anti–guinea pig IgG (A21450; Thermo Fisher Scientific), together with DAPI (D3571; Invitrogen), all diluted in blocking solution. The following day, samples were washed with PBS and imaged using a spinning disk confocal microscope (Leica DMi8, Leica Microsystems) equipped with a 10× objective.

Statistical analysis

All statistical analyses were performed using GraphPad Prism. Before analysis, surface tension and fluorescence intensity values were log10-transformed, as the raw data consistently followed log-normal distributions across conditions. This transformation improved normality and allowed the use of parametric statistical tests. One-way ANOVA followed by appropriate multiple-comparison post hoc tests was used for all group comparisons. Analyses were performed separately for each dataset, including surface tension measurements (Fig. 1F-H), CAF spatial organization metrics (Fig. 6B), and EMT-related fluorescence ratios (Fig. 6D). For each case, full pairwise comparisons were computed, with selected biologically relevant comparisons reported in Supplementary Tables and full outputs provided in the Source Data file. Statistical significance is reported as ns ($p \geq 0.05$), * ($p < 0.05$), ** ($p < 0.01$), *** ($p < 0.001$), and **** ($p < 0.0001$).

Supplementary Tables

	surface tension (mN/m)	
**breast CAF**	**48,7**	*± 16,9*
**pancreatic CAF**	**63,4**	*± 26,4*
**melanoma CAF**	**76,4**	*± 35,9*
**Hs578T**	**62,7**	*± 35,2*
**MCF7**	**2,4**	*± 1,1*
**T47D**	**1,8**	*± 1,1*
**MDA-MB-231**	**0,7**	*± 0,4*
**hPAF**	**14,8**	*± 9,7*
**PANC1**	**2,3**	*± 1,3*
**MiaPaCa**	**0,7**	*± 0,2*
**A375**	**0,4**	*± 0,2*
**Hs578T-CAF**	**61,7**	*± 18,5*
**MCF7-CAF**	**6,0**	*± 3,3*
**T47D-CAF**	**4,0**	*± 3,1*
**MDA-CAF**	**13,4**	*± 4,3*
**hPAF-CAF**	**29,2**	*± 12,5*
**PANC1-CAF**	**10,1**	*± 5,2*
**MiaPaCa-CAF**	**5,3**	*± 3,7*
**A375-CAF**	**12,7**	*± 6,5*

Table S1: Mean surface tension values measured for all 19 experimental conditions. Surface tensions are reported in mN/m ± standard deviation, obtained from magnetic force tensiometry measurements. The dataset includes three CAF spheroid conditions corresponding to breast, pancreatic, and melanoma origins. The next eight entries correspond to tumor spheroids formed from cancer cell lines alone (Hs578T, MCF7, T47D, MDA-MB-231, hPAF, PANC-1, MiaPaCa, and A375). The final eight entries correspond to heterotypic tumor–CAF spheroids formed by co-aggregation of each cancer cell line with its corresponding CAF population (Hs578T–CAF, MCF7–CAF, T47D–CAF, MDA-MB-231–CAF, hPAF–CAF, PANC-1–CAF, MiaPaCa–CAF, and A375–CAF).

Tukey's multiple comparisons test	Mean Diff	95,00% CI of diff	Summary	P Value
Breast CAF vs. Hs578T	-0,1233	-0,3742 to 0,1276	ns	0,9654
Breast CAF vs. Hs578T-CAF	-0,1199	-0,4076 to 0,1678	ns	0,9939
Breast CAF vs. MCF7	1,309	1,063 to 1,555	****	<0,0001
Breast CAF vs. MCF7-CAF	0,9177	0,6890 to 1,146	****	<0,0001
Breast CAF vs. T47D	1,431	1,186 to 1,676	****	<0,0001
Breast CAF vs. T47D-CAF	1,125	0,8643 to 1,386	****	<0,0001
Breast CAF vs. MDA-MB-231	1,83	1,547 to 2,113	****	<0,0001
Breast CAF vs. MDA-CAF	0,5598	0,2672 to 0,8525	****	<0,0001
Hs578T vs. Hs578T-CAF	0,003335	-0,2644 to 0,2710	ns	>0,9999
Hs578T vs. MCF7	1,432	1,210 to 1,655	****	<0,0001
Hs578T vs. MCF7-CAF	1,041	0,8380 to 1,244	****	<0,0001
Hs578T vs. T47D	1,554	1,334 to 1,775	****	<0,0001
Hs578T vs. T47D-CAF	1,249	1,010 to 1,487	****	<0,0001
Hs578T vs. MDA-MB-231	1,953	1,690 to 2,216	****	<0,0001
Hs578T vs. MDA-CAF	0,6831	0,4101 to 0,9561	****	<0,0001
Hs578T-CAF vs. MCF7	1,429	1,166 to 1,692	****	<0,0001
Hs578T-CAF vs. MCF7-CAF	1,038	0,7906 to 1,285	****	<0,0001
Hs578T-CAF vs. T47D	1,551	1,289 to 1,813	****	<0,0001
Hs578T-CAF vs. T47D-CAF	1,245	0,9680 to 1,522	****	<0,0001
Hs578T-CAF vs. MDA-MB-231	1,95	1,652 to 2,248	****	<0,0001
Hs578T-CAF vs. MDA-CAF	0,6798	0,3726 to 0,9869	****	<0,0001
MCF7 vs. MCF7-CAF	-0,3915	-0,5885 to -0,194	****	<0,0001
MCF7 vs. T47D	0,122	-0,09344 to 0,337	ns	0,8819
MCF7 vs. T47D-CAF	-0,1839	-0,4177 to 0,0499	ns	0,3476
MCF7 vs. MDA-MB-231	0,5209	0,2625 to 0,7793	****	<0,0001
MCF7 vs. MDA-CAF	-0,7493	-1,018 to -0,4807	****	<0,0001
MCF7-CAF vs. T47D	0,5135	0,3183 to 0,7088	****	<0,0001
MCF7-CAF vs. T47D-CAF	0,2076	-0,007677 to 0,42	ns	0,0736
MCF7-CAF vs. MDA-MB-231	0,9124	0,6706 to 1,154	****	<0,0001
MCF7-CAF vs. MDA-CAF	-0,3578	-0,6105 to -0,105	***	0,0001
T47D vs. T47D-CAF	-0,3059	-0,5383 to -0,073	***	0,0007
T47D vs. MDA-MB-231	0,3988	0,1418 to 0,6559	****	<0,0001
T47D vs. MDA-CAF	-0,8714	-1,139 to -0,6040	****	<0,0001
T47D-CAF vs. MDA-MB-231	0,7048	0,4322 to 0,9774	****	<0,0001
T47D-CAF vs. MDA-CAF	-0,5655	-0,8478 to -0,283	****	<0,0001
MDA-MB-231 vs. MDA-CAF	-1,27	-1,573 to -0,9672	****	<0,0001

Table S2. Pairwise statistical comparisons of surface tension measurements within the breast cancer dataset. Surface tension values were first log10-transformed, and statistical analysis was performed using one-way ANOVA followed by multiple-comparison testing. Only intra-breast cancer comparisons are reported here, while the full set of 171 pairwise comparisons is provided in the Source Data file (sheet: “Fig.1-stat”). The first column lists the two conditions compared. The second column reports the mean difference in log10(surface tension), with positive values indicating higher surface tension in the first condition and negative values indicating higher surface

tension in the second. The third column shows the 95% confidence interval of the difference, and the fourth column provides the significance summary (ns, *, **, ***,), with corresponding adjusted p-values reported in the fifth column (corresponding to $p < 0.0001$).

Tukey's multiple comparisons test	Mean Diff	95,00% CI of diff	Summary	P Value
Pancreatic CAF vs. hPAF	0,734	0,4387 to 1,029	****	<0,0001
Pancreatic CAF vs. hPAF-CAF	0,4105	0,03873 to 0,7823	*	0,0143
Pancreatic CAF vs. PANC1	1,543	1,208 to 1,877	****	<0,0001
Pancreatic CAF vs. PANC1-CAF	0,8955	0,5455 to 1,246	****	<0,0001
Pancreatic CAF vs. MiaPaCa	2,002	1,616 to 2,388	****	<0,0001
Pancreatic CAF vs. MiaPaCa-CAF	1,208	0,8219 to 1,594	****	<0,0001
hPAF vs. hPAF-CAF	-0,3235	-0,6724 to 0,0254	ns	0,1074
hPAF vs. PANC1	0,8085	0,5000 to 1,117	****	<0,0001
hPAF vs. PANC1-CAF	0,1615	-0,1641 to 0,4871	ns	0,9622
hPAF vs. MiaPaCa	1,268	0,9037 to 1,632	****	<0,0001
hPAF vs. MiaPaCa-CAF	0,4739	0,1098 to 0,8380	***	0,0009
hPAF-CAF vs. PANC1	1,132	0,7496 to 1,514	****	<0,0001
hPAF-CAF vs. PANC1-CAF	0,485	0,08862 to 0,8813	**	0,0028
hPAF-CAF vs. MiaPaCa	1,591	1,163 to 2,020	****	<0,0001
hPAF-CAF vs. MiaPaCa-CAF	0,7974	0,3689 to 1,226	****	<0,0001
PANC1 vs. PANC1-CAF	-0,647	-1,008 to -0,2858	****	<0,0001
PANC1 vs. MiaPaCa	0,4593	0,06302 to 0,8555	**	0,0069
PANC1 vs. MiaPaCa-CAF	-0,3346	-0,7309 to 0,0616	ns	0,2255
PANC1-CAF vs. MiaPaCa	1,106	0,6966 to 1,516	****	<0,0001
PANC1-CAF vs. MiaPaCa-CAF	0,3124	-0,09732 to 0,722	ns	0,4066
MiaPaCa vs. MiaPaCa-CAF	-0,7939	-1,235 to -0,3530	****	<0,0001

Tukey's multiple comparisons test	Mean Diff	95,00% CI of diff	Summary	P Value
Melanoma CAF vs. A375	2,113	1,731 to 2,496	****	<0,0001
Melanoma CAF vs. A375-CAF	0,7462	0,4003 to 1,092	****	<0,0001
A375 vs. A375-CAF	-1,367	-1,749 to -0,9846	****	<0,0001

Tukey's multiple comparisons test	Mean Diff	95,00% CI of diff	Summary	P Value
Breast CAF vs. Pancreatic CAF	-0,2147	-0,5128 to 0,0833	ns	0,5182
Breast CAF vs. Melanoma CAF	-0,1448	-0,4560 to 0,1664	ns	0,9799
Pancreatic CAF vs. Melanoma CAF	0,06997	-0,2642 to 0,4041	ns	>0,9999

Table S3. Pairwise statistical comparisons of surface tension measurements for the pancreatic and melanoma datasets, including comparisons between the three CAF populations. Surface tension values were first log10-transformed, and statistical analysis was performed using one-way ANOVA followed by multiple-comparison testing. Only intra-group comparisons are reported here, while the full set of 171 pairwise comparisons is provided in the Source Data file (sheet: “Fig.1-stat”). The first column lists the two conditions compared. The second column reports the mean difference in log10(surface tension), with positive values indicating higher surface tension

in the first condition and negative values indicating higher surface tension in the second. The third column shows the 95% confidence interval of the difference, the fourth column provides the significance summary (ns, *, **, ***,), and the fifth column reports the adjusted p-value corresponding to p < 0.0001).

	IC50-1	IC50-1	IC50-1	**IC50 (µM)**	
**BreastCAF 2D**	0,9355	1,042	0,4653	**0,81**	± *0.31*
**BreastCAF 3D**	2,187	1,917	0,5488	**1,55**	± *0.88*
**PancCAF 2D**	1,898	0,9193	0,6067	**1,14**	± *0.67*
**PancCAF 3D**	1,413	0,2656	3,655	**1,78**	± *1.72*
**Hs578T 2D**	0,5557	0,9865	1,339	**0,96**	± *0.39*
**Hs578T 3D**	2,162	6,572	3,368	**4,03**	± *2.28*
**Hs578T@CAF**	1,833	1,05	2,379	**1,75**	± *0.67*
**MCF7 2D**	1,997	0,5707	2,207	**1,59**	± *0.89*
**MCF7 3D**	0,8748	2,352	1,51	**1,58**	± *0.74*
**MCF7@CAF**	0,9421	2,955	1,054	**1,65**	± *1.13*
**MDA-MB-231 2D**	0,6464	1,39	0,898	**0,98**	± *0.38*
**MDA-MB-231 3D**	0,3634	1,308	2,105	**1,26**	± *0.87*
**MDA@CAF**	0,8735	2,597	4,135	**2,54**	± *1.63*
**hPAF 2D**	4,38	6,512	6,86	**5,92**	± *1.34*
**hPAF 3D**	8,929	5,287	5,195	**6,47**	± *2.13*
**hPAF@CAF**	1,793	3,729	3,915	**3,15**	± *01.18*
**PANC1 2D**	0,1171	0,2612	0,1713	**0,18**	± *0.07*
**PANC1 3D**	1,695	0,4468	0,5342	**0,89**	± *0.70*
**PANC1@CAF**	1,096	0,6775	1,634	**1,14**	± *0,48*
**MiaPaCa 2D**	0,1582	0,1011	0,1203	**0,13**	± *0.03*
**MiaPaCa 3D**	0,7419	0,2892	0,5991	**0,54**	± *0.23*
**MiaPaCa@CAF**	1,372	0,7682	1,938	**1,36**	± *0,59*

Table S4: IC50 values for paclitaxel determined in 2D adherent cultures, 3D homotypic spheroids, and 3D heterotypic cancer–CAF spheroids. Values are reported for each independent biological experiment, together with the mean ± standard deviation across the three experiments. IC50 values were obtained from nonlinear regression of normalized dose–response curves as shown in Supplementary Figs. S6–S8.

Tukey's multiple comparisons test	Mean Diff	95,00% CI of diff	Summary	P Value
Hs578T **mixCAF** vs. Hs578T **sph@CAF**	-0,7589	-1,009 to -0,5086	****	<0,0001
Hs578T **mixCAF** vs. MCF7**mixCAF**	0,5806	0,3303 to 0,8308	****	<0,0001
Hs578T **mixCAF** vs. MCF7**sph@CAF**	0,6444	0,3942 to 0,8947	****	<0,0001
Hs578T **mixCAF** vs. T47D**mixCAF**	0,5956	0,3453 to 0,8458	****	<0,0001
Hs578T **mixCAF** vs. T47D**sph@CAF**	0,8867	0,6364 to 1,137	****	<0,0001
Hs578T **mixCAF** vs. MDA**mixCAF**	-0,007222	-0,2575 to 0,2431	ns	>0,9999
Hs578T **mixCAF** vs. MDA**sph@CAF**	0,1233	-0,1270 to 0,3736	ns	0,9442
Hs578T **sph@CAF** vs. MCF7**mixCAF**	1,339	1,089 to 1,590	****	<0,0001
Hs578T **sph@CAF** vs. MCF7**sph@CAF**	1,403	1,153 to 1,654	****	<0,0001
Hs578T **sph@CAF** vs. T47D**mixCAF**	1,354	1,104 to 1,605	****	<0,0001
Hs578T **sph@CAF** vs. T47D**sph@CAF**	1,646	1,395 to 1,896	****	<0,0001
Hs578T **sph@CAF** vs. MDA**mixCAF**	0,7517	0,5014 to 1,002	****	<0,0001
Hs578T **sph@CAF** vs. MDA**sph@CAF**	0,8822	0,6319 to 1,133	****	<0,0001
MCF7**mixCAF** vs. MCF7**sph@CAF**	0,06389	-0,1864 to 0,3142	ns	>0,9999
MCF7**mixCAF** vs. T47D**mixCAF**	0,015	-0,2353 to 0,2653	ns	>0,9999
MCF7**mixCAF** vs. T47D**sph@CAF**	0,3061	0,05582 to 0,5564	**	0,0033
MCF7**mixCAF** vs. MDA**mixCAF**	-0,5878	-0,8381 to -0,3375	****	<0,0001
MCF7**mixCAF** vs. MDA**sph@CAF**	-0,4572	-0,7075 to -0,2069	****	<0,0001
MCF7**sph@CAF** vs. T47D**mixCAF**	-0,04889	-0,2992 to 0,2014	ns	>0,9999
MCF7**sph@CAF** vs. T47D**sph@CAF**	0,2422	-0,008072 to 0,4925	ns	0,0698
MCF7**sph@CAF** vs. MDA**mixCAF**	-0,6517	-0,9020 to -0,4014	****	<0,0001
MCF7**sph@CAF** vs. MDA**sph@CAF**	-0,5211	-0,7714 to -0,2708	****	<0,0001
T47D**mixCAF** vs. T47D**sph@CAF**	0,2911	0,04082 to 0,5414	**	0,0073
T47D**mixCAF** vs. MDA**mixCAF**	-0,6028	-0,8531 to -0,3525	****	<0,0001
T47D**mixCAF** vs. MDA**sph@CAF**	-0,4722	-0,7225 to -0,2219	****	<0,0001
T47D**sph@CAF** vs. MDA**mixCAF**	-0,8939	-1,144 to -0,6436	****	<0,0001
T47D**sph@CAF** vs. MDA**sph@CAF**	-0,7633	-1,014 to -0,5130	****	<0,0001
MDA**mixCAF** vs. MDA**sph@CAF**	0,1306	-0,1197 to 0,3808	ns	0,9131
hPAF**mixCAF** vs. hPAF**sph@CAF**	-2,19	-2,440 to -1,940	****	<0,0001
hPAF**mixCAF** vs. PANC1**mixCAF**	-0,2878	-0,5381 to -0,03748	**	0,0086
hPAF**mixCAF** vs. PANC1**sph@CAF**	-0,1361	-0,3864 to 0,1142	ns	0,8825
hPAF**mixCAF** vs. MiaPaCa**mixCAF**	-0,6911	-0,9414 to -0,4408	****	<0,0001
hPAF**mixCAF** vs. MiaPaCa**sph@CAF**	-0,5317	-0,7820 to -0,2814	****	<0,0001
hPAF**sph@CAF** vs. PANC1**mixCAF**	1,902	1,652 to 2,153	****	<0,0001
hPAF**sph@CAF** vs. PANC1**sph@CAF**	2,054	1,804 to 2,304	****	<0,0001
hPAF**sph@CAF** vs. MiaPaCa**mixCAF**	1,499	1,249 to 1,749	****	<0,0001
hPAF**sph@CAF** vs. MiaPaCa**sph@CAF**	1,658	1,408 to 1,909	****	<0,0001
PANC1**mixCAF** vs. PANC1**sph@CAF**	0,1517	-0,09863 to 0,4020	ns	0,7663
PANC1**mixCAF** vs. MiaPaCa**mixCAF**	-0,4033	-0,6536 to -0,1530	****	<0,0001
PANC1**mixCAF** vs. MiaPaCa**sph@CAF**	-0,2439	-0,4942 to 0,006405	ns	0,0653
PANC1**sph@CAF** vs. MiaPaCa**mixCAF**	-0,555	-0,8053 to -0,3047	****	<0,0001
PANC1**sph@CAF** vs. MiaPaCa**sph@CAF**	-0,3956	-0,6458 to -0,1453	****	<0,0001
MiaPaCa**mixCAF** vs. MiaPaCa**sph@CAF**	0,1594	-0,09085 to 0,4097	ns	0,6942
A375**mixCAF** vs. A375**sph@CAF**	0,1389	-0,1114 to 0,3892	ns	0,865

Table S5: Pairwise statistical comparisons of CAF spatial organization (OUT/IN or LEFT/RIGHT metrics) across heterotypic cancer–CAF conditions shown in Figures 4 and 5. Analyses were performed using one-way ANOVA with multiple comparisons on log10-transformed values. Results are grouped by cancer type (breast, pancreatic, melanoma), while the full set of pairwise

comparisons is provided in the Source Data file (sheet: “Fig6B-stat”). For each comparison, values include mean difference, 95% confidence interval, significance summary, and adjusted p-value.

Tukey's multiple comparisons test	Mean Diff	95,00% CI of diff	Summary	P Value
BreastCAF vs. HS578T	0,02257	-0,2429 to 0,2881	ns	>0,9999
BreastCAF vs. MCF7	1,527	1,252 to 1,801	****	<0,0001
BreastCAF vs. T47D	1,804	1,526 to 2,081	****	<0,0001
BreastCAF vs. MDA-MB-231	-0,3035	-0,5810 to -0,02602	*	0,019
HS578T vs. MCF7	1,504	1,254 to 1,754	****	<0,0001
HS578T vs. T47D	1,781	1,528 to 2,034	****	<0,0001
HS578T vs. MDA-MB-231	-0,3261	-0,5787 to -0,07343	**	0,0018
MCF7 vs. T47D	0,2771	0,01476 to 0,5394	*	0,0284
MCF7 vs. MDA-MB-231	-1,83	-2,092 to -1,568	****	<0,0001
T47D vs. MDA-MB-231	-2,107	-2,372 to -1,842	****	<0,0001
PancreaticCAF vs. hPAF	1,7	1,429 to 1,970	****	<0,0001
PancreaticCAF vs. PANC1	0,6485	0,3767 to 0,9202	****	<0,0001
PancreaticCAF vs. MiaPaCa	-0,0875	-0,3593 to 0,1843	ns	0,9941
hPAF vs. PANC1	-1,051	-1,315 to -0,7874	****	<0,0001
hPAF vs. MiaPaCa	-1,787	-2,051 to -1,523	****	<0,0001
hPAF vs. MelanomaCAF	-1,849	-2,119 to -1,578	****	<0,0001
PANC1 vs. MiaPaCa	-0,736	-1,001 to -0,4707	****	<0,0001
MelanomaCAF vs. A375	0,4185	0,1508 to 0,6861	****	<0,0001
BreastCAF vs. PancreaticCAF	-0,005499	-0,2893 to 0,2783	ns	>0,9999
BreastCAF vs. MelanomaCAF	-0,1545	-0,4383 to 0,1293	ns	0,8023
PancreaticCAF vs. MelanomaCAF	-0,149	-0,4272 to 0,1292	ns	0,8181

Table S6: Pairwise statistical comparisons of normalized Vimentin, E-cadherin, and Vimentin-to-E-cadherin ratio across all cancer cell lines and CAF populations. Analyses were performed using one-way ANOVA with multiple comparisons on log10-transformed values. Results are grouped by cancer type, while the full set of pairwise comparisons is provided in the Source Data file (sheet: “Fig6D-stat”). For each comparison, values include mean difference, 95% confidence interval, significance summary, and adjusted p-value.

Client Sample Name	PANC-1	MiaPaCa	MCF7	Hs578T
Sample Code	CL00017305	CL00017306	CL00017307	CL00017302
D8S1179	14,15	16,16	10,14	13,13
D21S11	28,28	29,31.2	30,30	29,32.2
D7S820	8,10	12,13	8,9	10,10
CSF1PO	10,12	10,10	10,10	13,13
D3S1358	17,17	16,16	16,16	16,17
TH01	7,8	9,10	6,6	9,9.3
D13S317	11,11	12,13	11,11	11,11
D16S539	11,11	10,13	11,12	9,12
D2S1338	23,24	25,25	21,23	17,26
D19S433	11,16	15,15	13,14	14,15
vWA	15,15	15,15	14,15	17,17
TPOX	8,11	9,9	9,12	8,8
D18S51	12,12	12,12	14,14	16,16
AMEL	X,X	X,X	X,X	X,X
D5S818	11,13	12,13	11,12	11,11
FGA	21,21	22,22	23,25	23,24
**Database Name**	PANC-1	MIA-PACA-2	MCF-7	Hs 578T

Client Sample Name	A375	hPAF	T47D	MDA
Sample Code	CL00017299	CL00017301	CL00017296	CL00017290
D8S1179	11,14	11,12	13,13	13,13
D21S11	29,30	30,31	28,31	30,33.2
D7S820	9,9	10,13	11,11	8,9
CSF1PO	11,12	10,11	11,13	12,13
D3S1358	15,17	14,18	15,17	16,16
TH01	8,8	9,9	6,6	7,9.3
D13S317	11,14	12,12	12,12	13,13
D16S539	9,9	11,13,14	10,10	12,12
D2S1338	16,24	16,19	24,24	20,21
D19S433	13,14.2	10,12	14,14	11,14
vWA	16,17	17,17	14,14	15,18
TPOX	8,10	8,8	11,11	8,9
D18S51	12,17	13,13	17,17	11,16
AMEL	X,X	X,X	X,X	X,X
D5S818	12,12	11,13	12,12	12,12
FGA	23,23	21,24	23,23	22,23
**Database Name**	A-375	HPAF-II*	T47D	MDA-MB-231

Table S7. STR profiling of all cancer cell lines. Short tandem repeat (STR) profiling was performed by Eurofins Genomics Europe Food/Environment/White Biotech Products & Services GmbH (Anzinger Str. 7, D-85560 Ebersberg, Germany) to authenticate all cancer cell lines prior to use. Genomic DNA was isolated from cell pellets and analyzed using PCR-single-locus technology. Sixteen independent STR loci were evaluated: D8S1179, D21S11, D7S820, CSF1PO, D3S1358, TH01, D13S317, D16S539, D2S1338, D19S433, vWA, TPOX, D18S51, D5S818, FGA, and the sex-determining marker AMEL. Core markers (ASN-0002) are indicated in grey and were analyzed using the AmpFlSTR® Identifiler® Plus PCR Amplification Kit (Thermo Fisher Scientific). Positive and negative controls were included in each run and yielded expected results. STR profiles were matched against reference databases provided by the cell line repository to confirm identity. Full analytical outputs, including sample identifiers, allele calls for each locus, and database matching information, are provided in the table. All experimental procedures followed the manufacturer's standard operating protocol SOP_APG_Zelllinienauthentizität_3.0_15.04.2024.

Supplementary Figures

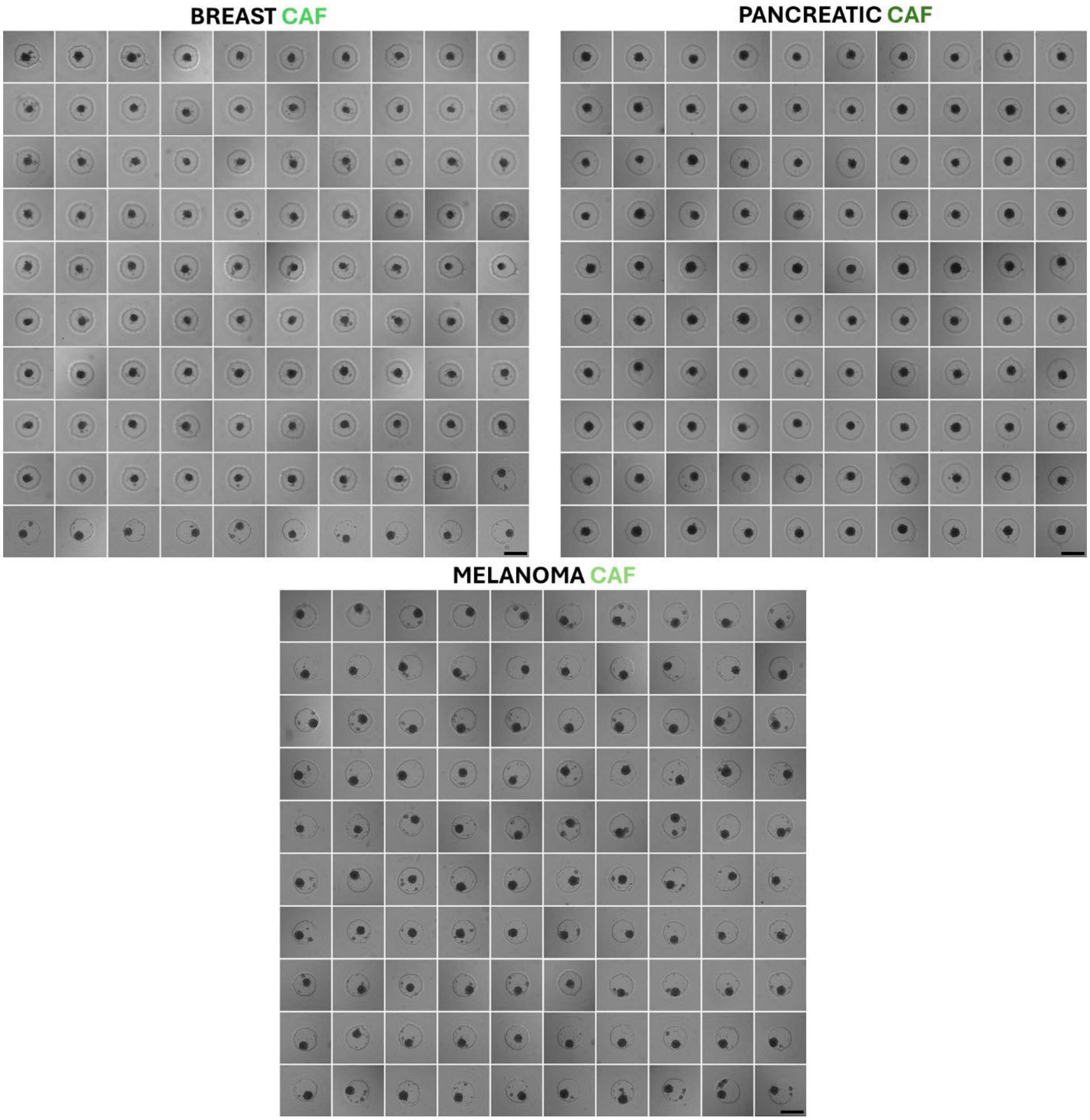

Figure S1: Representative brightfield images of spheroids formed in 500 µm agarose microwells two days after seeding 2,000 cells per microwell. CAF-only spheroids formed from breast, pancreatic, and melanoma CAF populations. Scale bar, 500 µm.

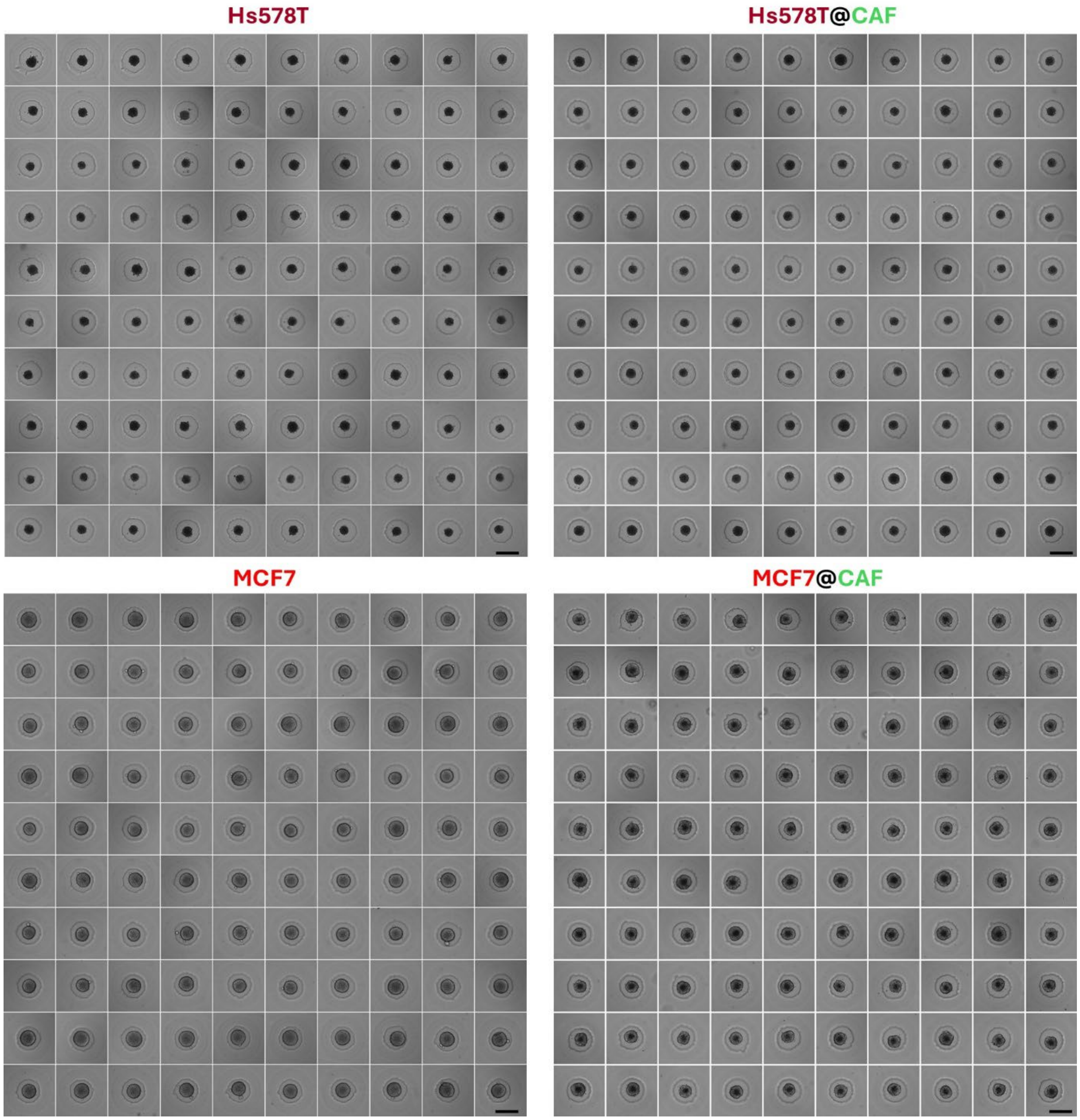

Figure S2: Representative brightfield images of Hs578T and MCF7 spheroids formed in 500 µm agarose microwells, in the absence or presence of CAFs (cancer:CAF ratio 70:30), two days after seeding 2,000 cells per microwell. Scale bar, 500 µm.

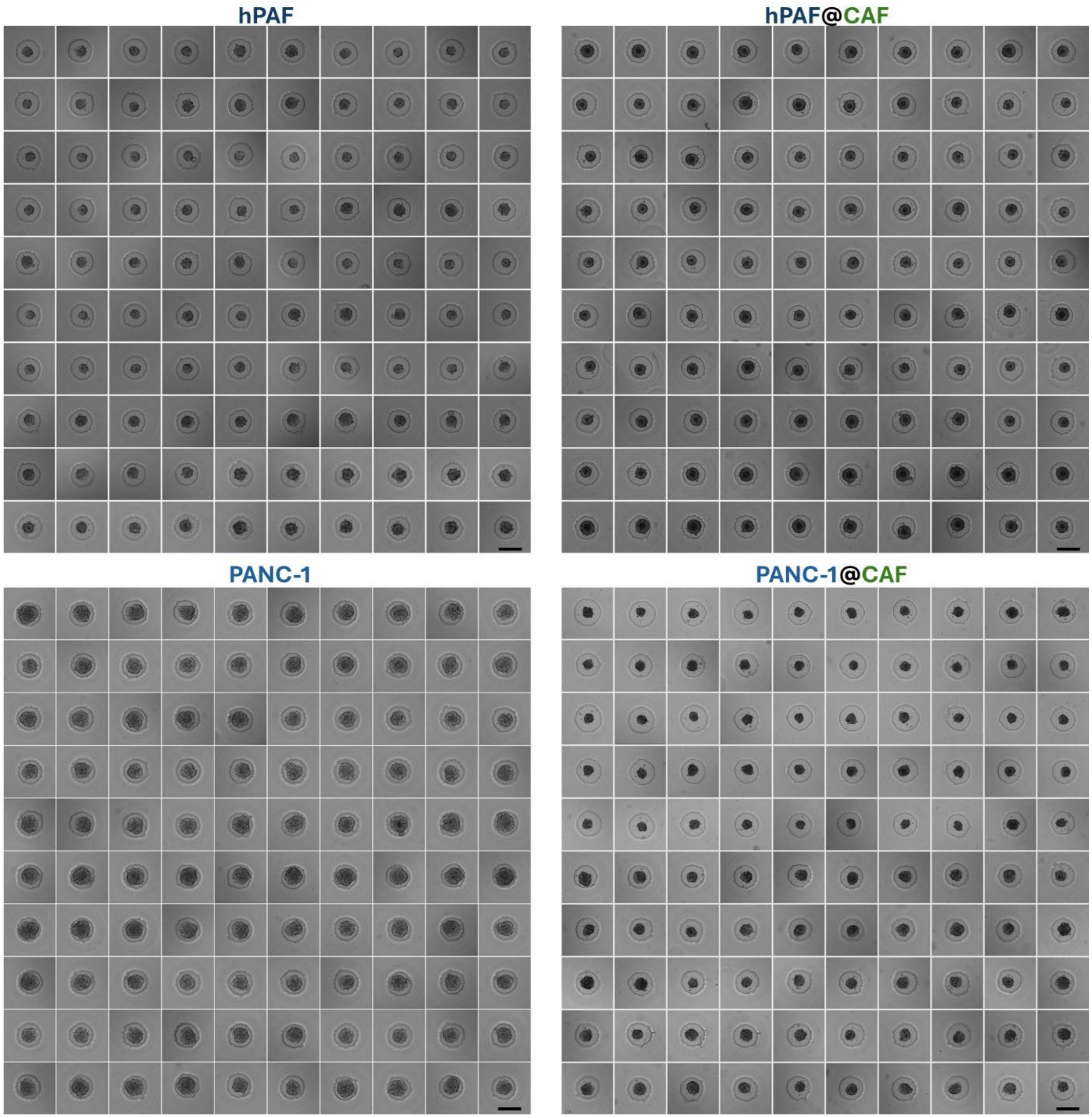

Figure S3: Representative brightfield images of hPAF and PANC-1 spheroids formed in 500 µm agarose microwells, in the absence or presence of CAFs (cancer:CAF ratio 70:30), two days after seeding 2,000 cells per microwell. Scale bar, 500 µm.

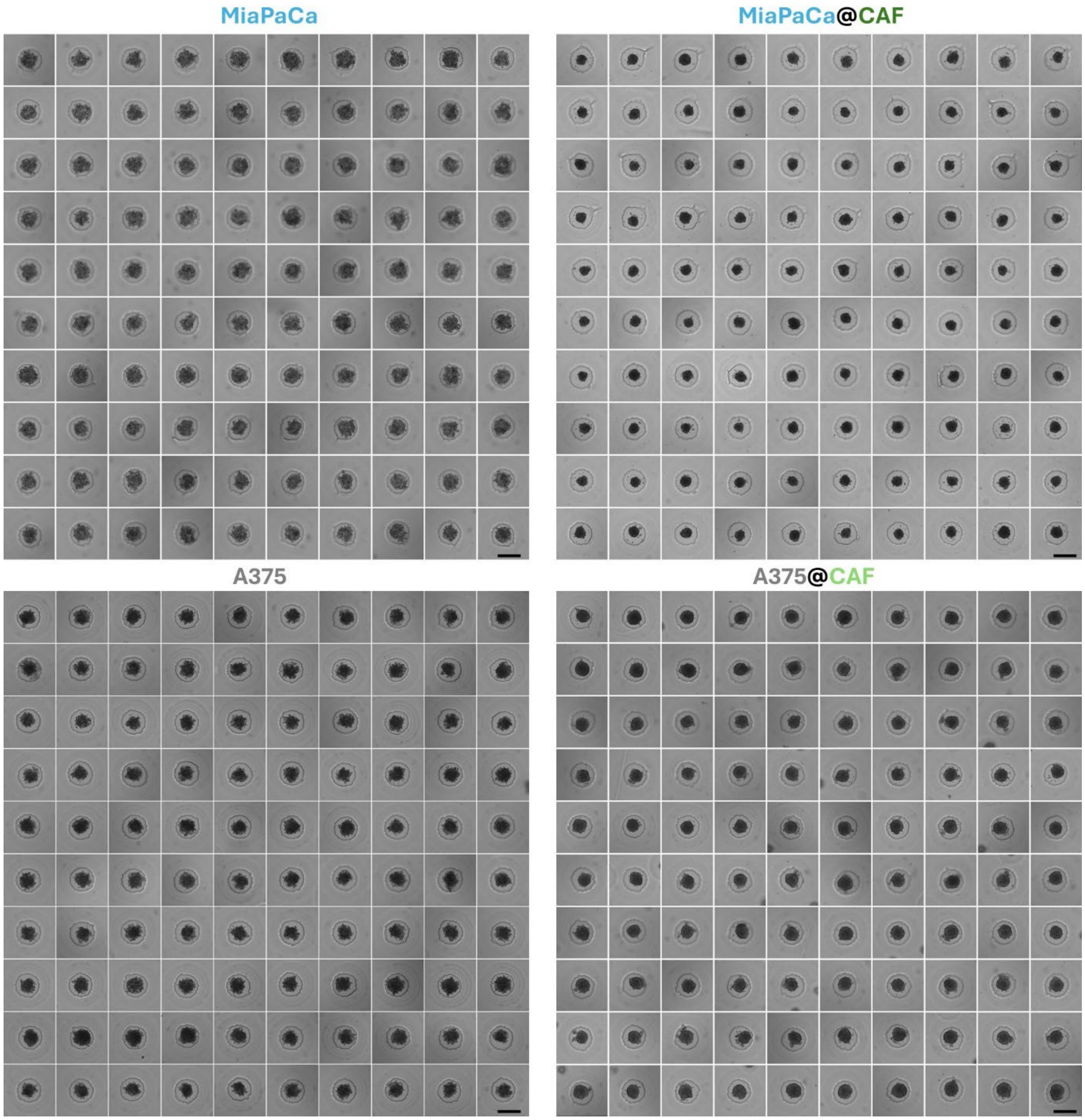

Figure S4: Representative brightfield images of MiaPaCa and A375 spheroids formed in 500 µm agarose microwells, in the absence or presence of CAFs (cancer:CAF ratio 70:30), two days after seeding 2,000 cells per microwell. Scale bar, 500 µm.

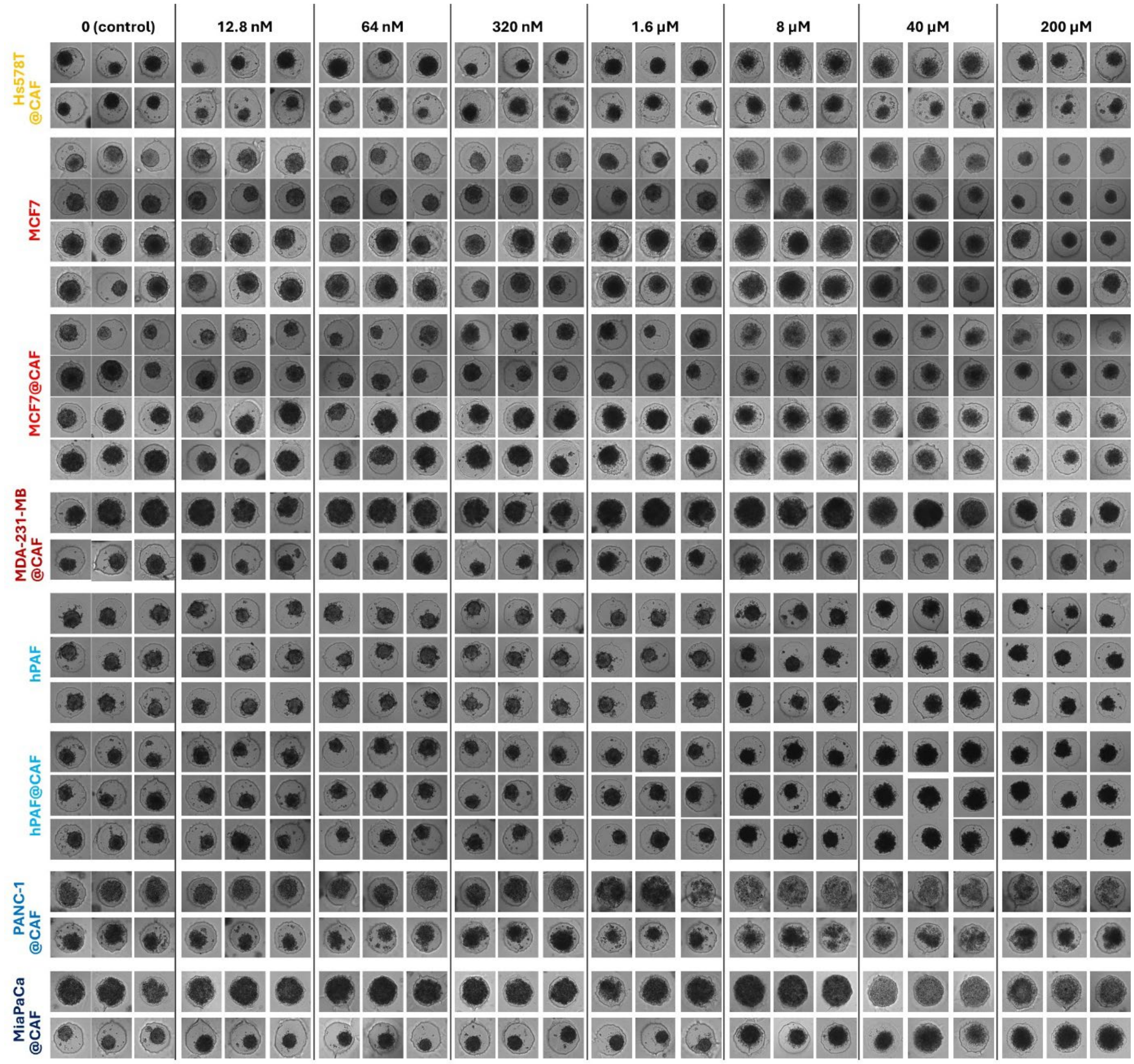

Figure S5: Representative endpoint images after two days of paclitaxel treatment for Hs578T, MCF7, MDA-MB-231, hPAF, PANC-1, and MiaPaCa spheroids. Columns correspond to paclitaxel concentrations, including untreated controls and increasing doses from 12.8 nM to 200 µM. Each row corresponds to a cell line, with two sub-rows showing spheroids cultured without CAFs (top) and with corresponding CAFs (70:30, bottom), with three representative spheroids shown per condition. Scale bar, 500 µm.

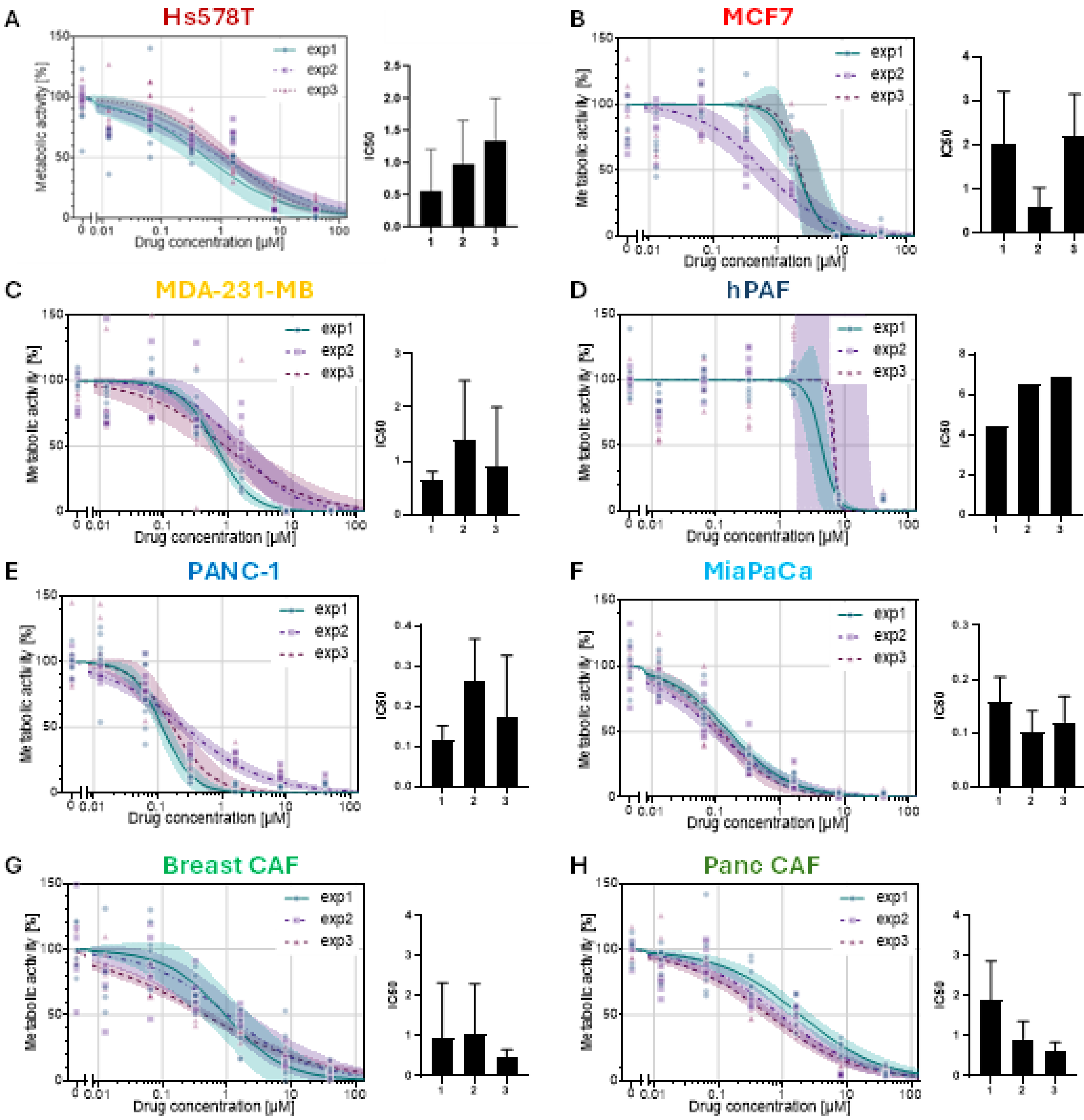

Figure S6: Dose–response curves and IC50 determination for paclitaxel in 2D adherent cultures. Each panel corresponds to one experimental condition across the six tumor cell lines and the two CAF populations (eight panels total): A = Hs578T, B = MCF7, C = MDA-MB-231, D = hPAF, E = PANC-1, F = MiaPaCa, G = breast CAFs, H = pancreatic CAFs. Individual data points represent single wells, with full dose–response measurements shown for each independent biological experiment. Three independent experiments are represented using distinct markers: experiment 1 (green circles), experiment 2 (purple squares), and experiment 3 (dark red triangles). For each experiment, IC50 values were obtained by nonlinear regression using the standard model in GraphPad Prism ([Inhibitor] vs. normalized response). Final IC50 values are summarized in left-hand insets as bar plots showing each mean IC50 (± error) across the three independent experiments (1, 2, and 3).

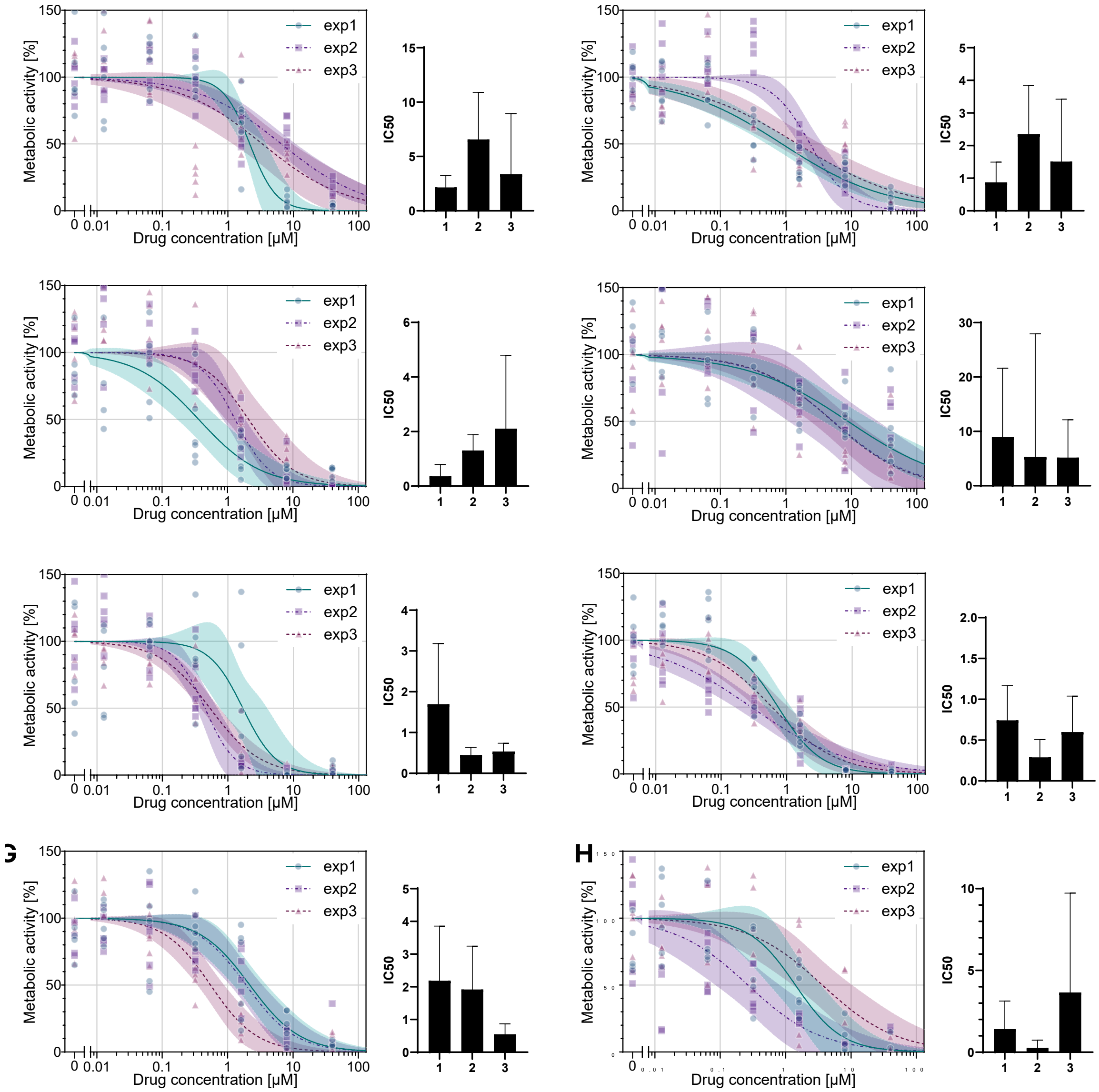

Figure S7: Dose–response curves and IC50 determination for paclitaxel in 3D spheroid cultures. Experimental layout, cell line assignment (A–H), biological replicates, and data representation are identical to Supplementary Fig. S6, but performed in 3D conditions. Individual data points represent single wells, with full dose–response measurements shown for each independent biological experiment. Three independent experiments are represented using distinct markers: experiment 1 (green circles), experiment 2 (purple squares), and experiment 3 (dark red triangles). IC50 values were obtained by nonlinear regression using GraphPad Prism ([Inhibitor] vs. normalized response). Final IC50 values are summarized in left-hand insets as bar plots showing each mean IC50 (± error) across the three independent experiments (1, 2, and 3).

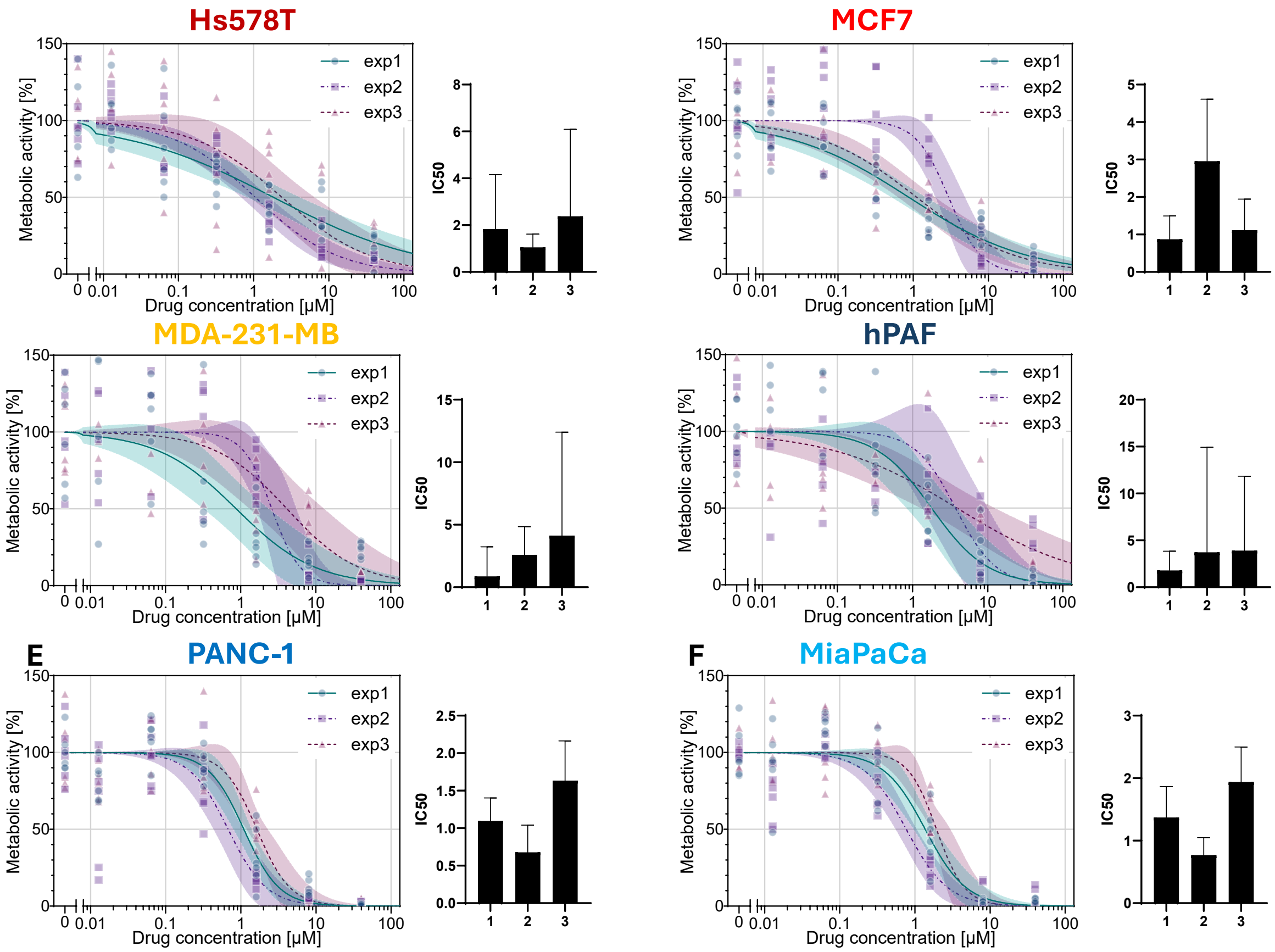

Figure S8: Dose–response curves and IC50 determination for paclitaxel in 3D heterotypic cancer–CAF spheroids (70:30). Each panel corresponds to one across the six tumor cell lines: A = Hs578T, B = MCF7, C = MDA-MB-231, D = hPAF, E = PANC-1, F = MiaPaCa. Individual data points represent single wells, with full dose–response measurements shown for each independent biological experiment. Three independent experiments are represented using distinct markers: experiment 1 (green circles), experiment 2 (purple squares), and experiment 3 (dark red triangles). IC50 values were obtained by nonlinear regression using GraphPad Prism ([Inhibitor] vs. normalized response). Final IC50 values are summarized in left-hand insets as bar plots showing each mean IC50 (± error) across the three independent experiments (1, 2, and 3).

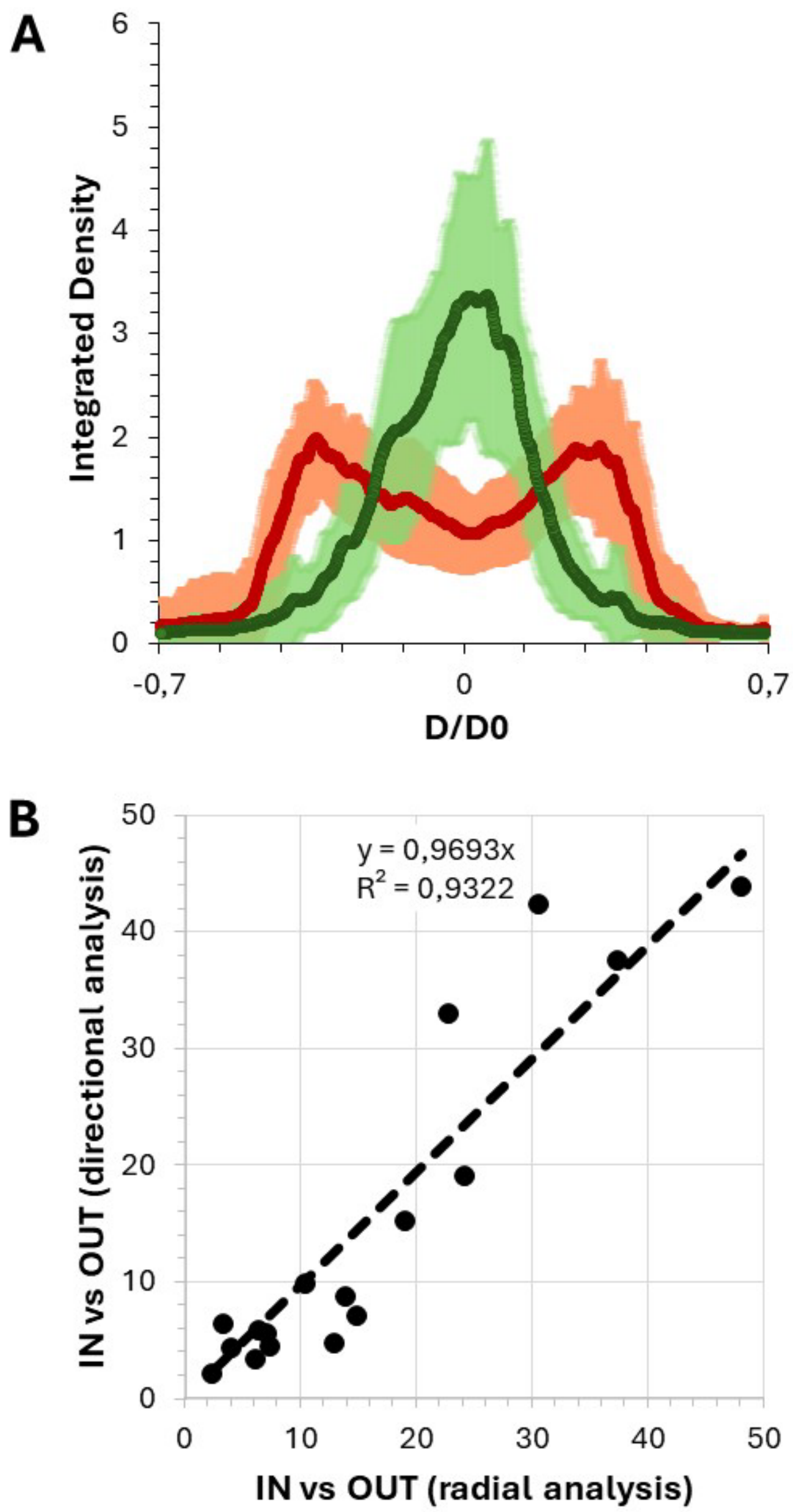

Figure S9: **Validation of longitudinal versus radial analysis in non-dumbbell-like spheroids.** **A**: Longitudinal fluorescence distribution for T47D spheroids in sequential cancer–CAF assembly conditions (T47D sph@CAF, as shown in Fig. 5). Analysis was performed using the same directional framework as for hPAF spheroids exhibiting dumbbell-like morphology in Fig. 5, in which fluorescence intensity is quantified along opposing axes instead of radial averaging. The curve represents the mean profile obtained from 18 independent spheroids. Individual spheroid measurements and full longitudinal profiles are provided in the Source Data file (sheet "Fig5", longitudinal analysis section). **B**: Correlation between CAF localization metrics obtained using radial (CAF IN/OUT) and longitudinal analysis approaches. Each point corresponds to an independent spheroid. The two methods show agreement, with a correlation coefficient > 0.93.

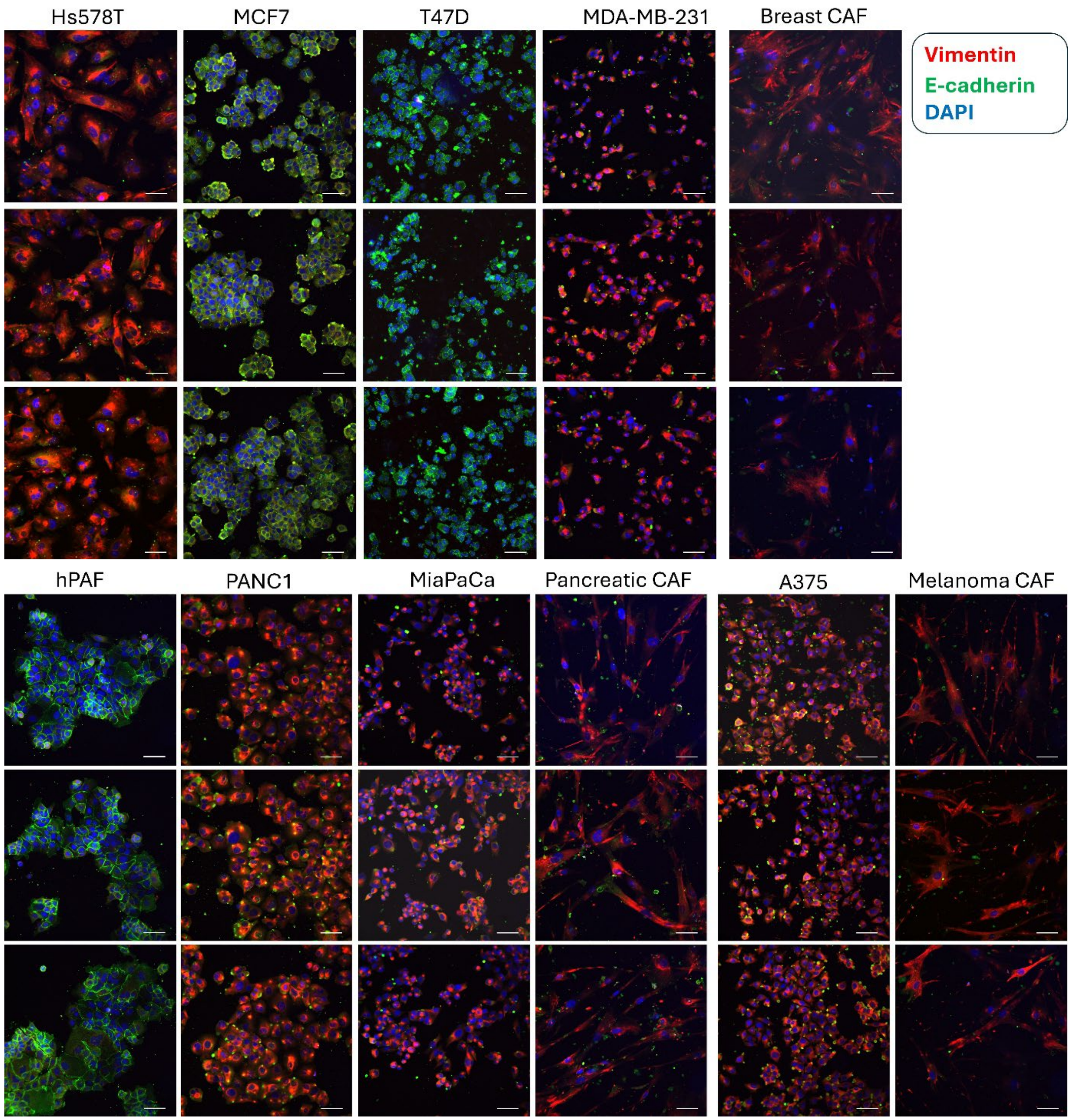

Figure S10: Additional representative immunofluorescence images of Vimentin (red), E-cadherin (green), and nuclei (DAPI, blue) for all tumor cell lines and CAF populations (11 conditions total). Images were acquired under identical exposure and thresholding settings as those used in Figure 6C. Scale bar, 100 µm.

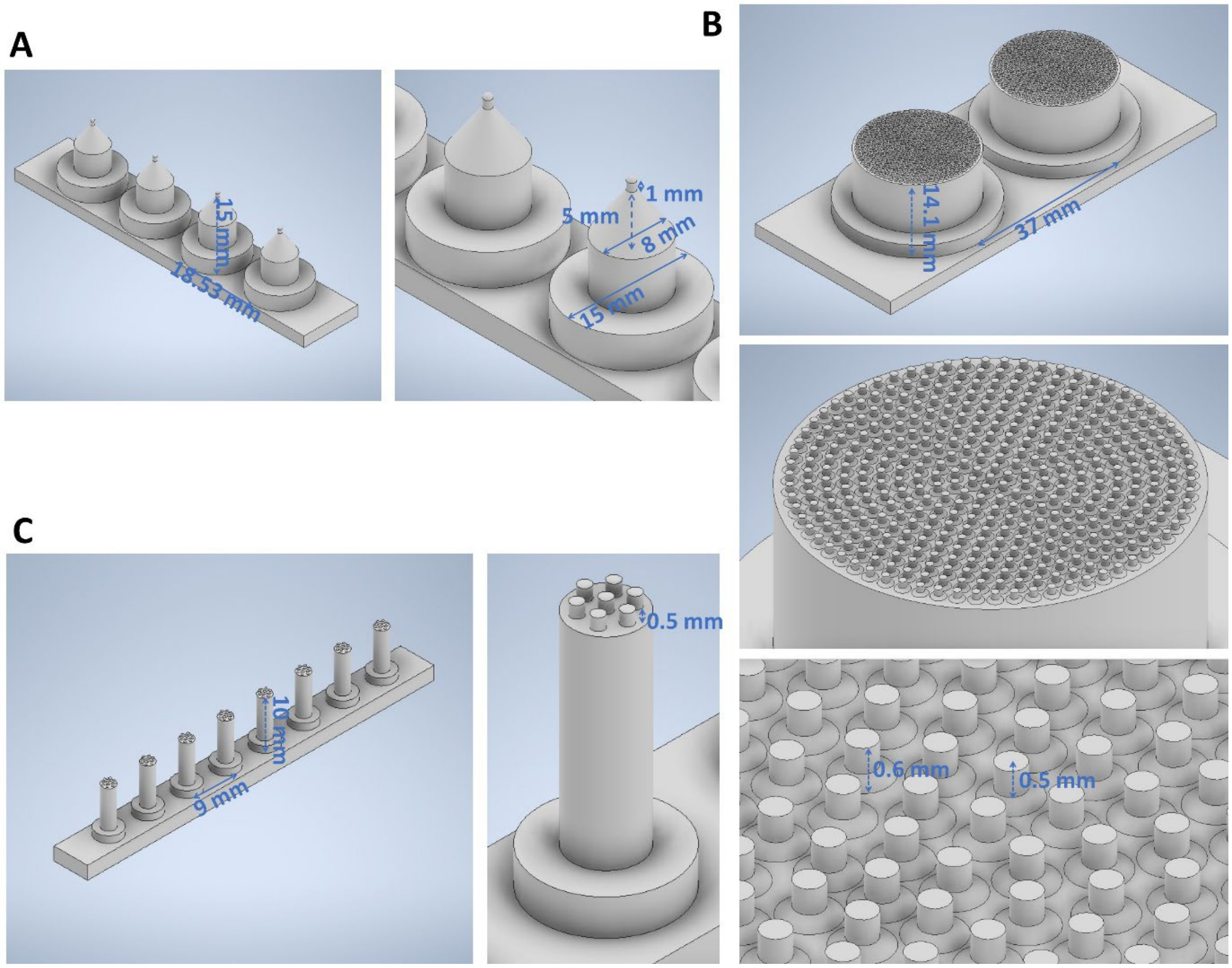

Figure S11: **Design and fabrication of 3D-printed microwell molds.** Schematic representation of the three custom-designed microwell geometries. All molds were fabricated in-house using a DigitalWax 028J Plus 3D printer (DWS Systems) with LOCTITE 3D MED412 biocompatible resin (ISO 10993-5 and ISO 10993-10 certified) at a spatial resolution of 25 × 25 µm. Three configurations were used: (**A**) 24-well format with a single central microwell (1 mm diameter) for magnetic force tensiometry (Figure 1); (**B**) 6-well format containing 401 microwells per well (500 µm diameter, 500 µm depth) for large-scale spheroid production (Figures 2, 4, 5); and (**C**) 96-well format with 7 microwells per well (500 µm diameter, 500 µm height) for drug response assays (Figure 3). All molds feature conical pillar tops to facilitate cell capture.
